# Long-read sequencing reveals the landscape of aberrant alternative splicing and novel therapeutic target in colorectal cancer

**DOI:** 10.1186/s13073-023-01226-y

**Published:** 2023-09-21

**Authors:** Qiang Sun, Ye Han, Jianxing He, Jie Wang, Xuejie Ma, Qianqian Ning, Qing Zhao, Qian Jin, Lili Yang, Shuang Li, Yang Li, Qiaoming Zhi, Junnian Zheng, Dong Dong

**Affiliations:** 1 Cancer Institute, Xuzhou Medical University, 209 Tongshan Road, Xuzhou, 221004 Jiangsu Province China; 2 Center of Clinical Oncology, the Affiliated Hospital of Xuzhou Medical University, Jiangsu, Xuzhou, China; 3 Jiangsu Center for the Collaboration and Innovation of Cancer Biotherapy, Cancer Institute, Xuzhou Medical University, 209 Tongshan Road, Jiangsu, Xuzhou, 221004 China; 4 Future Health Laboratory, Innovation Center of Yangtze River Delta, Zhejiang University, Jiashan, 314100, China; 5 Department of General Surgery, The First Affiliated Hospital of Soochow University, Suzhou, China; 6 International Institutes of Medicine, The Fourth Affiliated Hospital, Zhejiang University School of Medicine, Yiwu, 322000 China

**Keywords:** Long-read sequencing, Alternative splicing, *TIMP1 Δ4-5*, Colorectal cancer

## Abstract

**Background:**

Alternative splicing complexity plays a vital role in carcinogenesis and cancer progression. Improved understanding of novel splicing events and the underlying regulatory mechanisms may contribute new insights into developing new therapeutic strategies for colorectal cancer (CRC).

**Methods:**

Here, we combined long-read sequencing technology with short-read RNA-seq methods to investigate the transcriptome complexity in CRC. By using experiment assays, we explored the function of newly identified splicing isoform *TIMP1 Δ4-5.* Moreover, a CRISPR/dCasRx-based strategy to induce the *TIMP1* exon 4–5 exclusion was introduced to inhibit neoplasm growth.

**Results:**

A total of 90,703 transcripts were identified, of which > 62% were novel compared with current transcriptome annotations. These novel transcripts were more likely to be sample specific, expressed at relatively lower levels with more exons, and oncogenes displayed a characteristic to generate more transcripts in CRC. Clinical outcome data analysis showed that 1472 differentially expressed alternative splicing events (DEAS) were tightly associated with CRC patients’ prognosis, and many novel isoforms were likely to be important determinants for patient survival. Among these, newly identified splicing isoform *TIMP1 Δ4-5* was significantly downregulated in CRC. Further in vitro and in vivo assays demonstrated that ectopic expression of *TIMP1 Δ4-5* significantly suppresses tumor cell growth and metastasis. Serine/arginine-rich splicing factor 1 (SRSF1) acts as a onco-splicing regulator through sustaining the inclusion of *TIMP1* exon 4–5. Furthermore, CRISPR/dCasRx-based strategies designed to induce *TIMP1* exon 4–5 exclusion have the potential to restrain the CRC growth.

**Conclusions:**

This data provides a rich resource for deeper studies of gastrointestinal malignancies. Newly identified splicing isoform *TIMP1 Δ4-5* plays an important role in mediating CRC progression and may be a potential therapy target in CRC.

**Supplementary Information:**

The online version contains supplementary material available at 10.1186/s13073-023-01226-y.

## Background

Alternative splicing (AS) is one of the most important mechanisms of posttranscriptional regulation, with over 90% of human multi-exon genes alternatively spliced [1, 2]. It provides the potential to generate diversity at RNA and protein levels, which largely enriches the cellular protein reservoir and contributes to temporal and spatial diversification of biological function [3, 4]. Besides being a critical mechanism during development and cell differentiation, alternative splicing is involved in multiple oncological processes, including angiogenesis, invasion and metastasis [5, 6]. Aberrant AS has been considered a hallmark of cancer, and systematic study of AS may provide potential biomarkers for malignancies.

Colorectal cancer (CRC) is one of the most common malignancies with a high mortality rate [7, 8]. Mounting evidence has unveiled that AS alterations are harbored within CRC [9–12]. Aberrant AS has also been implicated in CRC carcinogenesis. For example, *ZO-1* pre-mRNA is alternatively spliced into two variants, *ZO-1* E23 + and *ZO-1* E23 − , encoding protein isoforms with different biological effects. Downregulation of *ZO-1* E23 + contributed to the tumorigenesis and metastasis, whereas *ZO-1* E23 − variant plays no functional role in tumor metastasis [9]. To determine the significance of AS in CRC, comprehensively describing the AS landscape and their biological/clinical implications is necessary.

To date, systematic identification of AS remains a challenging task [13]. The transcriptome complexity has been largely undermined because of technical limitations in systematic analysis. During the past decades, short RNA sequencing reads (≤ 150 nt) have played a leading role in transcriptome annotation [14, 15]. However, RNA-seq using short reads encounters severe limitations in reconstructing the full-length transcript and identifying isoforms with complex AS events. Obviously, transcriptome using long-read sequencing would be ideal for elucidating the AS landscape and the identification of full-length splice isoforms [16–18]. Recently, Oxford Nanopore Technologies (ONT) has been widely used to analyze full-length splicing isoforms [19, 20]. Despite a relatively high error rate of the ONT long sequencing reads, this problem can be resolved by integrating matched short-read sequencing data using computational methods [21, 22].

In this study, a comprehensive AS landscape in CRC was detected using ONT long-read sequencing and Illumina short-read sequencing platforms. Thousands of unannotated spliced isoforms were identified and some of these novel transcripts were tightly associated with patients’ survival, which largely broaden our understanding of CRC tumorigenesis. Among these newly identified splicing isoforms, *TIMP1 Δ4-5* transcript (exon 4–5 exclusion of tissue inhibitor matrix metalloproteinase 1) was significantly downregulated in CRC and suppresses tumor cell growth and metastasis. It has been documented that the full-length *TIMP1* (*TIMP1-FL*) is an oncogenic transcript and plays a pivotal role in CRC tumorigenesis [23]. Therefore, mediating splicing isoform switch from *TIMP1-FL* to *TIMP1 Δ4-5* would be necessary for CRC therapy. Mutations in the nuclease domain of CasRx can generate a catalytic ‘dead’ enzyme that retains RNA-binding affinity (dCasRx). It has been documented that recruitment of CRISPR/dCasRx systems (nuclease-inactive RNA targeting CasRx) to regulatory splice sites can induce isoform-switching splicing changes by interfering with the recruitment of splicing machinery [24]. To this end, a CRISPR/dCasRx strategy was employed to induce *TIMP1* exon 4–5 exclusion in vitro and in vivo, showing promising therapeutic value of targeting *TIMP1* alternative splicing in CRC.

## Methods

### Patient specimens

A total of 78 pairs of CRC cancer and adjacent normal tissues were collected from patients who undergone surgery between September 2012 and February 2014 at the Department of General Surgery, the First Affiliated Hospital of Soochow University (Suzhou, China). Patient samples were collected with informed consent from patients, and experiments were approved by the ethics committees of the First Affiliated Hospital of Soochow University. Tissue samples were snap-frozen in liquid nitrogen and stored at − 80 °C after surgical resection. All of the specimens were verified by histopathology and none of the patients were received preoperative chemotherapy or radiotherapy. Cohort details are provided in Additional file 2: Table S1.

### RNA sequencing

A total of 78 CRC tumor tissues and 10 adjacent normal tissues were selected for RNA sequencing. Total RNAs were isolated using Qiagen R Neasy Mini Kit. RNA integrity and concentration were tested using Eukaryote total RNA Nano assay on BioAnalyzer (CHOP NAPCore). For long-read sequencing, Oxford MinION 1D amplicon libraries were generated according to the Oxford Nanopore community protocol and sequenced on R9 flow cells according to the manufacturer’s protocol. For short-read sequencing, total RNAs were subjected to ribosomal RNA removal using the Ribo-Zero™ Magnetic Kit (Epicentre Technologies). Truseq Stranded RNA sequencing libraries were prepared with 500 ng total RNA according to the manufacturer’s protocol (Illumina). The libraries were sequenced in a 2 × 150 nt paired-end manner on Nova-Seq 6000 platform (Illumina) according to the manufacturer’s protocol.

### Long-read sequencing data quality control, alignment, and correction

Base-calling of MinION FAST5 reads was performed using albacore v1.1.0 and Guppy v3.3.0 with default parameters, and porechop v0.2.4 was used to trim the adapter of the ONT reads. Only reads passed the quality control by Nanofilter v2.8.0 were retained for subsequent analyses. The ONT reads were aligned to human reference genome hg38 using minimap2 v2.2.14 [25] in spliced alignment mode with the command ‘mnimap2 -ax splice’. The obtained reads were mapped to human genome using Minimap2 through “split-read” alignment. The ONT sequencing data statistics were performed with NanoStat v1.5.0. FLAIR *correct* module v1.5.0 was adopted to correct the splice-site boundaries of ONT reads [22]. All splice sites were evaluated for validity by checking whether they were supported by GENCODE v34 annotations or Illumina sequencing short reads. Spliced junctions were extracted from corresponding short reads, and only the junctions with ≥ 3 uniquely mapping short reads were considered valid. The corrected long reads were made up of reads with accurate splice sites supported by short reads. For transcripts containing novel splicing junctions, we mapped the short reads to the novel transcripts and ≥ 3 short-read support means that the transcripts have short-read sequencing support. For intergenic, intronic, and single-exon transcripts, if more than 3 short reads uniquely mapped to the transcripts, then the transcripts were considered as short-read sequencing supported.

### Long-read sequencing transcript assembly

We used FLAIR collapse module v1.5.0 [22] to assemble transcripts based on the above corrected long reads with the default settings. Briefly, the assembling processes consisted of the following steps: (1) The TSSs and TTSs were determined by the density of the long-read start and end coordinates to construct the first-pass assembly. (2) 100-nt windows of end sites of each read were compared to find the most frequent site in the window (parameter: -n best_only). (3) The final ONT reference assembly was determined by aligning raw reads to the first-pass assembly using minimap2, retaining only the first-pass transcripts with ≥ 3 supporting reads with high alignment quality (MAPQ ≥ 1). As a result, a non-redundant merged transcriptome based on 78 CRC and 10 adjacent normal samples was created for further analysis.

### Long-read sequencing transcript annotation and quality control

Our ONT long-read transcript annotation pipeline utilizing several tools was delineated in Additional file 1: Fig. S1. SQANTI3 v1.6.1 [26] was used to annotate the splice transcripts based on GENCODE v34. The SQANTI3 (https://github.com/ConesaLab/SQANTI3) also provided a list of quality attributes including CAGE peaks, poly(A) sites, NMD prediction and so on to help filter out potential artifacts and getting high-quality transcript annotation. Briefly, SQANTI3 was used to generate an indel-corrected GTF or FASTA files by realigning the transcripts obtained from the FLAIR pipeline to the human reference genome hg38, as well as to categorize transcripts into several types (FSM, NIC, NNC, ISM, antisense, genic, fusion, and intergenic) based on their splicing patterns based on GENCODE v34. The gene fusions were detected by Fusionseeker v1.0.1 under the default parameters [27].

Transcripts were filtered based on the following criteria to ensure their validity: (1) all splice junctions were supported by ≥ 5 short reads; (2) the transcript was detected in at least 10% samples (≥ 9 samples) based on long-read ONT data; (3) transcripts with unreliable 3’ end based on the following standards: ≥ 100 bp away from the annotated TTSs, having no overlap with the poly(A) site database which integrating 3’-seq data and curated poly(A) motif. Transcript-level or gene-level expression with transcripts per million (TPM) were calculated for each transcript or gene by Salmon v0.10.0 [28] for long-read data and StringTie v2.1.2 [29] for short-read data based on the reference CRC transcriptome generated by long-read sequencing data.

### Protein-level characterizations of long-read sequencing transcript

The transdecoder v5.5.0 was utilized to extract ORFs from transcripts, and the minimal length of ORF to be at least 300 bp [30]. The obtained ORF sequences were aligned to the UniProt (release 2020_12) database using blastp v2.11.0 with E-value < 1e − 5. Moreover, we also mapped the predicted ORF sequences to the PFAM v31.0 database with hmmer v3.2.1 using default options [31]. As a result, the single best ORFs for each transcript were selected based on blastp and hmmer results. The phyloP and phastCons scores of novel transcripts were calculated using bigWigAverageOverBed v377 with phyloP and phastCons data of 100 vertebrate species from UCSC, respectively.

We downloaded raw MS/MS data of CRC cancer patients (97 tumors, 100 adjacent normal tissues) from the CPTAC database (https://cptac-data-portal.georgetown.edu/study-summary/S037). Peptide identification was performed using Maxquant v1.6.10.43 [32] with a comprehensive sequence dataset by integrating long-read predicted ORFs and curated human protein sequences from the Uniprot database. The following parameters were set for peptide searching: (1) Carbamidomethylation (C) was set as a fixed modification; (2) Oxidation (M), Deamidated (NQ), and Acetyl (K) were selected as variable modifications; (3) The peptide mass tolerances were set as < 20 and 4.5 ppm for the first search and main search, respectively. (4) The peptide length was set as ≥ 7 amino acids (AAs) and the maximum peptide mass was 4600 Da; (5) Only one missed cleavage site was allowed; (6) Both peptides and proteins with FDR > 0.01 were filtered. Finally, protein groups were classified into three groups, namely ONT_only (peptides mapped to ONT transcript and not mapped to UniProt proteins), ONT + Uniprot (peptides mapped to both ONT transcript and UniProt proteins), and Uniprot_only (peptides mapped to UniProt proteins and not mapped to ONT transcript), respectively. The DeepLoc [33] and TMHMM [34] were adopted to predict the subcellular localization and transmembrane domains, respectively.

### Short-read sequencing analysis

The quality control of short-read RNA-seq data was performed by FastQC v0.11.9 [35]. The low-quality reads and adapters were filtered and trimmed by using fastp v0.20.1 [36]. Then, all clean reads were mapped to the human reference genome hg38 using STAR v2.5.1 [37]. Specifically, a two-pass strategy was used to extract splice junctions from each sample. The short-read transcripts were assembled using StringTie v2.1.2 [29] with GENCODE v34 as reference.

### Alternative splicing event analysis

We used SUPPA2 v2.3 [38] to calculate 7 types of alternative splicing events (ASEs) including A3/A5 (alternative 3’ and 5’ splice sites), AF/AL (alternative first and last exons), SE (skipping exon), RI (retained intron), and MX (mutually exclusive exon) by using the assembled long-read CRC transcript annotations. Specifically, we used the *generateEvent* command in SUPPA2 with *–f ioe* options on the GTF file assembled from the FLAIR and SQANTI3 pipeline previously. Then, the PSI (percent spliced in) levels of each ASE were calculated using *psiPerEvent* command based on the transcript-level expression matrix generated by StringTie and the ioe file generated by *generateEvent.* The ASEs were further filtered to generate high-confidence events by retaining events with an average PSI value ≥ 0.05 in ≥ 75% of samples. To detect differentially spliced events, Wilcoxon singed-rank test followed by Benjamini–Hochberg correction was used to detect differentially spliced events between normal and tumor samples. DEAS events with adjusted* P-*value ≤ 0.05 and | *Δ*PSI|≥ 0.2 were considered to be statistically significant.

### Gene set and pathway enrichment analyses

The Hallmark gene sets which summarize and represent specific well-defined biological states or processes and display coherent expression are acquired from MsigDB [39]. The C2 curated gene sets which integrated online pathway databases and the biomedical literature were also acquired for further analysis. The R clusterProfiler v3.18.1 package [40] was used to assess pathway enrichment significance.

### Construction of dysregulation networks between SFs and DEAS events in CRC

The difference of expression of SFs between tumor and normal samples were calculated using DEseq2 v1.30.1 [41] with raw reads as input, and SFs with |log_2_(Fold Change)|≥ 1 and adjusted* P-*value ≤ 0.05 were considered as significantly expressed. We then performed Spearman correlation analyses between the expression level of SFs and the DEAS event expression; SF-AS event pairs with Spearman correlation coefficients ≥ 0.5 (or ≤  − 0.5) and adjusted* P-*value ≤ 0.05 were considered to be significantly correlated. Then, the dysregulation network was built and visualized by Cytoscape v3.8.2 software [42].

### Clinical outcome analysis

The univariate Cox model was used to evaluate the relation between DEAS events and CRC prognosis by R survival v3.2.7 package; DEAS events with adjust* P-*value ≤ 0.05 were considered significantly prognostic. Survival in patients with high or low DEAS events level was compared using the Kaplan–Meier method and the log-rank test. We predicted the CMS molecular subtypes of each patient based on the transcriptome data by R CMScaller v2.0.1 package [43]. The MSI status of patients was based on the transcriptome data by R PreMSIm v1.0 package [44]. We employed the R estimate v1.0.13 package to characterize the stromal and immune cell infiltration [45], and then analyzed the correlation between CRC microenvironment and splicing alterations in CRC samples under the threshold of adjusted *P*-value ≤ 0.05 and |Spearman correlation coefficient *r*|≥ 0.5.

#### Cell culture

Human CRC cell lines (SW620, SW480, LoVo, HCT-8, HCT116) were obtained from the American Type Culture Collection (ATCC, Manassas, VA, USA), and human CRC epithelial cell line NCM-460 was obtained from IN CELL (San Antonio, TX, USA). NCM-460, HCT-8, SW620, and SW480 were maintained in RPMI-1640 (Genom, China) medium containing 10% fetal bovine serum (FBS). HCT116 was cultured in McCoy’s 5A medium (Genom, China), LoVo was cultured in F-12 K medium (Genom, China), and all above medium were supplemented with 10% FBS (Gibco, USA) and penicillin/streptomycin. All cell lines were cultured at 37 °C in humidified 5% CO_2_ atmosphere. Cells were routinely tested and had negative results for mycoplasma.

### RNA isolation, reverse transcription, and qRT-PCR

Cell samples were lysed using TRIzol reagent (Invitrogen). Tissue samples were grounded to fine powder in liquid nitrogen and lysed using TRIzol reagent (Invitrogen). Total RNA was extracted according to the manufacturer’s protocol. Reverse transcription of RNA to cDNA was performed by reverse transcription polymerase chain reaction kit (Vazyme). qRT-PCR using SYBR Green (Vazyme) was performed on a LightCycler 480 instrument (Roche). The mRNA levels of human genes or mouse genes were assessed by qRT-PCR. Each qRT-PCR reaction was independently repeated at least three times to ensure reproducibility. The data were analyzed by 2^−ΔΔCT^ method. The primers for qRT-PCR were designed by online tool (Sigma) as indicated in Table S17.

### Western blotting

For western blotting, cells were harvested in cold PBS and lysed in cold radioimmunoprecipitation assay (RIPA) buffer (50 mM Tris at pH 7.5, 1% TritonX-100, 0.5% deoxycholate, 150 mM NaCl, 10 mM EDTA) containing protein inhibitor cocktail (Roche). Proteins were separated by SDS-PAGE and transferred to PVDF membranes. Membranes were incubated with primary antibodies and HRP-conjugated secondary antibody. The following antibodies were used: anti-human TIMP1 (1:1000, 16,644–1-AP, Proteintech), anti-human SRSF1 (1:1000, 12,929–2-AP, Proteintech), anti-human β-actin (1:1000, A5441, Sigma) and HRP-conjugated secondary antibody (1:5000, Thermo Scientific).

### Plasmids, siRNAs, and transfection

Full-length human TIMP1 and TIMP1 *Δ*4-5 were amplified from total RNA by RT-PCR and cloned into the pCS2 + vector. Small interfering RNAs (siRNAs) corresponding to the following sequences for knockdown of SRSF1 and TIMP1 *Δ*4-5 were synthesized by GenePharma (Shanghai, China): 5′-AGGACAUUGAGGACGUGUUTT-3′ for siSRSF1#l, 5′-GAAAGAAGAUAUGACCUAUTT -3′ for siSRSF1#2; 5′-UGGAUAAACAGGGAAACACCU-3′ for siTIMP1 *Δ*4-5. Lipofectamine 2000 reagent (Invitrogen, 11,668,019, USA) was used for transient transfection according to the manufacturer’s instructions.

### Construction of stable cell line

To generate cells stably expressing TIMP1 *Δ*4-5, TIMP1 *Δ*4-5 were amplified from cDNA and cloned into the pLVX-Puro vector. HEK293T cells were transfected with the indicated lentivirus expression vector and viral packaging constructs. Transient plasmid transfection was carried out with PolyJet (SignaGen Laboratories) DNA transfection reagent according to the manufacturer’s protocol. The viral medium was collected, filtered, and mixed with 1 μg/ml polybrene (Sigma) to infect the target cells. After 24 h infection, cells were treated with 1 mg/mL of puromycin (Thermo Scientific) for 1 week. The efficiency of upregulation was assessed by immunoblotting or qRT-PCR.

### Cell proliferation

For MTT assay, cancer cells were seeded in 96-well plates for the indicated times (0, 24, 48, 72, or 96 h), and relative cell number was measured 3-(4,5-Dimethylthiazol-2-yl)-2,5-diphenyltetrazolium bromide (Sigma) as described previously. For colony formation experiment, cancer cells were seeded in 35-mm-diameter dishes at a density of 1 × 10^3^ cells and incubated for 1 to 2 weeks until the colonies were visible to the naked eye. The colonies were stained with 0.5% crystal violet (Sigma) in 20% ethanol for 10 min and quantified by ImageJ software.

### Cell migration and invasion

For migration assay, 2.5 × 10^5^ cancer cells were suspended in 100 μl medium with 1% FBS were seeded in the upper chamber of transwell chambers (8 μm pores, Corning, USA), and 800 μl fresh medium with 10% FBS were added to the lower chamber. For invasion assay, 2.5 × 10^5^ cells were seeded in upper chamber pre-coated with Matrigel (8 μm pores, Corning, USA). After incubation for 24 h at 37 °C, the cells in the upper chamber were fixed with 4% paraformaldehyde, followed by staining with 0.1% crystal violet. The migrated or invaded cells were quantified by counting in five fields (100 ×) under an Olympus IX83 optical microscope (Olympus, Center Valley, USA). Each experiment was analyzed in triplicates.

### Generation of TIMP1 knockout cells by CRISPR/Cas9 system

To construct TIMP1 knockout cells by CRISPR/Cas9-mediated genome editing, a pair of targeting sequences (5′-AATTGCAGAAGGCCGTCTGT-3′) were cloned into our in-house modified one-vector system pEP330X as previously described. This plasmid was transfected into HCT-8 and SW480 cells for 48 h, and then the cells were seeded into 96-well plates for single-cell colony selection after 24 h treatment of 1 μg/mL puromycin. Following amplification of these colonies, individual genomic DNA was then extracted for Sanger sequencing analyses. The colonies with genome editing were further verified by western blotting with anti-TIMP1 antibody.

### Targeting TIMP1 AS by using dCasRx system

The dCasRx plasmid was obtained from Addgene (No. 109050, four residues in two HEPN-nuclease domains of CasRx was mutated as R239A/H244A/R858A/H863A). The CasRx gRNAs were golden-gate cloned into the pXR003-CasRx-gRNA (Addgene, No. 109053) with BbsI restriction enzyme and T4 ligase, which had constitutive gRNA expression driven by a U6 promoter. The competent cells used to amplify plasmids were DH5α (Vazyme).

The cas13design (https://cas13design.nygenome.org) is a flexible tool that is specifically developed to design optimal gRNAs with robust targeting efficiency for CasRx. Importantly, the cas13design has considered several features that are crucial for efficient RNA knockdown, including crRNA folding pattern and secondary structure of the target RNA. Thus, in order to achieve efficient exon exclusion of a TIMP1, we applied cas13design as the tool to design optimal gRNA for this study.

### RNA-binding protein immunoprecipitation (RIP)-qPCR

The CRC cells were washed twice with ice-cold PBS and lysed in 1 ml RIP Lysis buffer (150 mM KCl, 25 mM Tris, 5 mM EDTA, 0.5% Triton X-100, 0.5 mM DTT, Protease inhibitor (1:100), RNAase inhibitor (1:1000) on ice for 30 min. Cell lysates were centrifuged at 12,000 × *g* at 4 °C for 15 min. Ten percent supernatant was collected as input, and the remaining supernatant was incubated with anti-SRSF1 antibody or control IgG antibody that protein A/G conjugated magnetic beads (MCE, USA) in 900 μl RIP Lysis buffer at 4 °C for 4 h. Bound RNAs were immunoprecipitated with beads and wash beads with RIP buffer (150 mM KCl, 25 mM Tris, 5 mM EDTA, 0.5% Triton X-100) four times. Then the beads were treated with 10 μl 10% SDS, 10 μl Proteinase K, and 130 μl RIP buffer for 30 min at 55 °C. RNA in IP or Input group was recovered with Trizol reagent (Invitrogen, USA) according to the manufacturer’s instruction and analyzed by quantitative RT-PCR. IP enrichment ratio was calculated as a ratio of its amount in IP to that in the input.

### Animal models

All mice used in this study were approved by the Institutional Animal Care and Use Committee of Xuzhou Medical University. Female 4- to 6-week-old BALB/c nude mice were purchased from the GemPharmatech (Nanjing, China) and kept in SPF facilities (25 °C, suitable humidity (typically 50%), 12 h dark/light cycle), in which they can freely access to food and water. For xenograft tumor formation assay, SW480 cells (5 × 10^6^ cells/per mouse) were injected subcutaneously into mice as described previously. After 3 weeks, mice were randomly assigned to groups given either TIMP1 CRISPR/dCasRx vectors (4 μg for each mouse) plus polyethylenimine (Sigma-Aldrich, USA) delivery buffer or control delivery buffer via intratumoral injection twice per week, gross weight and tumor volume of the mouse were measured every 3 days until sacrificed at 7 weeks. Tumor volumes were measured by caliper and calculated by using the equation: *V* = (*L* × *W* × *W*)/2, where *V* is tumor volume (mm^3^), *L* is tumor length (mm), and *W* is tumor width (mm). The tumors were isolated, photographed, and weighed. After being fixed in 10% formaldehyde solution for 2 days, the mice tumors were embedded in paraffin and sectioned for further analyses.

## Results

### Characterization of CRC transcriptome using long-read and short-read sequencing methods

**Fig. 1:**
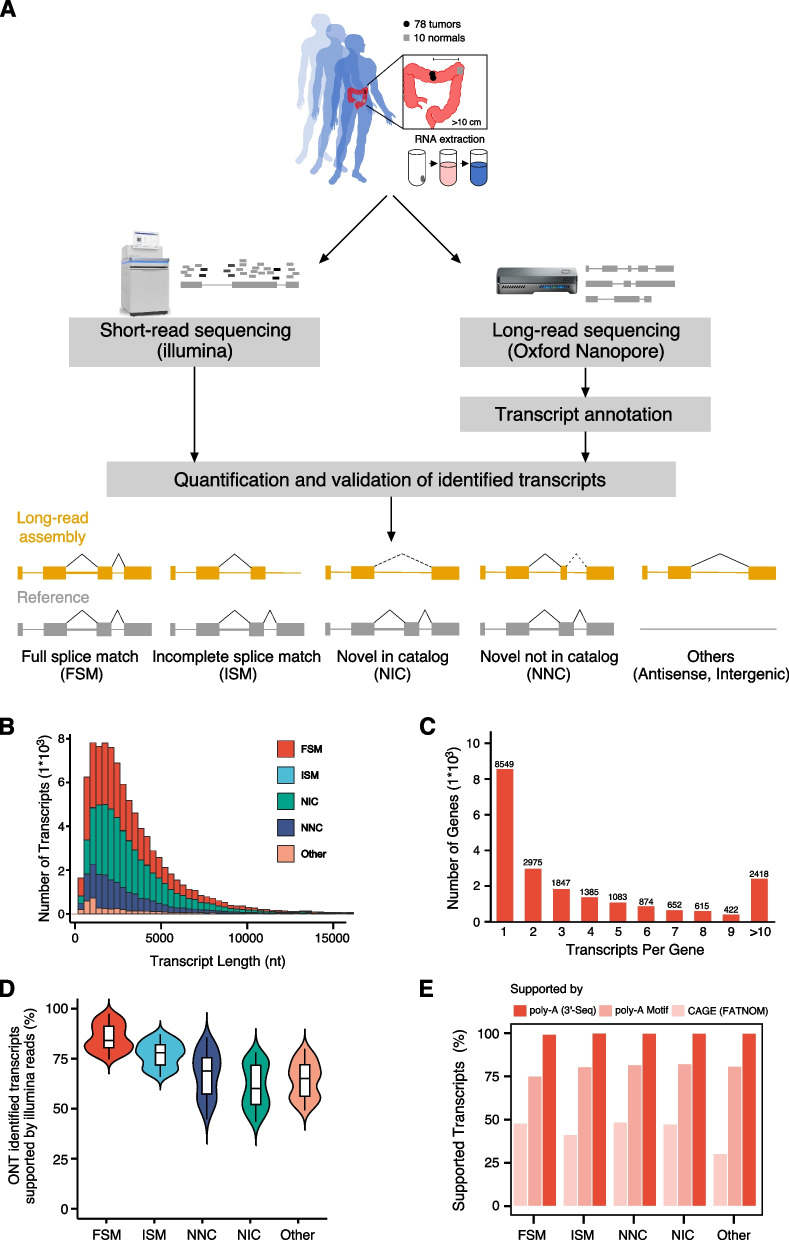
Long-read ONT sequencing identifies novel transcripts in CRC. **A** Workflow for transcriptome reconstruction based on ONT sequencing data. The FLAIR- SQANTI3 pipeline was adopted to assemble transcripts from long-read ONT data based on GENCODE v34. **B** Transcript length distribution of different types. **C** Number of transcripts identified per gene by ONT sequencing. **D** Percentage of long-read ONT transcripts supported by Short-Read Illumina RNA-seq in 78 CRC and 10 adjacent normal samples. **E** Percentage of long-read ONT transcripts’ TSSs supported by CAGE peaks, or TTSs supported by 3’-seq peaks or presence of a poly(A) motif

To characterize CRC transcripts with high confidence, we utilized both ONT long-read and Illumina short-read sequencing platforms to resolve CRC transcriptomes (Fig. 1A). Our CRC cohort consists of 78 primary human CRC and 10 adjacent normal biopsies (Additional file 2: Table S1). We obtained an average of 7.92 million ONT long reads with an average read length of 1.02 kb per sample, providing a reliable resource for exploring transcript diversity of human CRC transcriptomes (Table 1 and Additional file 2: Table S2). The schematic representation of the analytical pipeline was constructed (Additional file 1: Fig. S1). Over 90% of the long reads were mappable to the human genome, indicating their high specificity (Additional file 1: Fig. S2a). To validate the ability of ONT reads to recover full-length transcripts, we analyzed the percentage of full-length transcripts of human mitochondrial genes, which have a poly(A) tail and lack introns, resulting in fewer splicing complexities. The results revealed that nearly 60% of ONT reads of the mitochondrial genes were full-length, indicating that the ONT reads can recover most full-length transcripts that do not undergo alternative splicing (Additional file 1: Fig. S2b, c). The results demonstrated that these ONT reads can provide a reliable and valuable resource for analyzing CRC transcriptome complexity.

**Table 1:** ONT long-read sequencing statistics

	**Tumor tissues** **(*n* = 78)**	**Normal tissues (*n* = 10)**	**Overall** **(*n* = 88)**
**All reads**			
**Average read number (million)**	8.17	6.04	7.92
**Average length (kb)**	9.85	1.13	1.02
** Median length (kb)**	0.81	0.93	0.82
**N50 read length (kb)**	1.09	1.33	1.11
**Maximum read length (kb)**	1.89	7.69	2.55
A**verage read quality**	10.74	12.84	10.98
**Aligned reads**			
** Average read number (million)**	7.84	5.88	7.62
** Average length (kb)**	0.92	1.11	0.94
** Median length (kb)**	0.75	0.92	0.77
** N50 read length (kb)**	1.05	1.31	1.08
** Maximum read length (kb)**	18.46	76.90	25.10
** Average read quality**	10.81	12.84	11.04
** Average percent identity (%)**	89.96	92.82	90.29
** Median percent identity (%)**	90.68	94.03	91.06

The aligned reads generated by the ONT platform were then polished and assembled using the FLAIR pipeline [22], yielding an average of ~ 27,000 expressed genes with high confidence (Additional file 2: Table S3). Further quality control and transcript annotations were performed using SQANTI3 [26]. Finally, a total of 90,703 transcripts were identified based on a reference transcriptome (GENCODE v34) with a median transcript length of 2.5 kb (Fig. 1B and Additional file 1: Fig. S2d). These transcripts were mapped to 18,024 annotated genes and 2796 novel loci, and nearly 60% (12,271) of genes have two or more transcripts (Fig. 1C). Among the identified transcripts, 37% (33,913) were full-splice matches (FSM) perfectly matching known transcripts, and 40% (36,489), 17% (15,640), and 0.2% (215) were novel in catalog (NIC; refer to transcripts that containing new combinations of already annotated splice junctions or novel splice junctions formed from already annotated donors and acceptors), novel not in catalog (NNC; refer to transcripts that containing at least one novel junctions), and incomplete-splice matches (ISM; refer to transcripts that matching part of a known transcript), respectively (Additional file 1: Fig. S3a, b). In addition to these four transcript types, we also identified a fraction of other transcripts, such as antisense, genic, fusion, and intergenic types. However, we mainly focused on FSM, NIC, and NNC isoforms, which accounted for the vast majority (> 95%). Novel expressed transcripts (except FSM) account for 43 to 58% of all identified transcripts in individual samples (average = 53%, Additional file 1: Fig. S3a). The intriguing phenomenon is that about half of all identified transcripts are novel (NIC and NNC) in both tumor and normal samples (Additional file 1: Fig. S3b). We randomly selected several novel transcripts and validated them using qRT-PCT (Additional file 1: Fig. S3c). Overall, we identified thousands of novel transcripts in the CRC transcriptome that have not been annotated in the current reference.

To validate the reliability of long-read transcripts, we analyzed short-read RNA-seq data by mapping them to the long-read CRC transcriptome. We found that 84% of the FSM transcripts, 71% of the NIC transcripts, and 55% of the NNC transcripts were validated (Fig. 1D). Several NIC and NNC transcripts were validated using Sanger sequencing method (Additional file 1: Fig. S3d). We next used various orthogonal data to further assess the robustness of these transcripts, including CAGE (Cap Analysis Gene Expression, which measures the 5’ end of transcripts), 3’-seq (which measures the ploy(A) tail of transcripts), and some intrinsic sequencing properties. Benchmarking the novel transcripts against the FSM transcripts, we found that the 3’ ends of novel transcripts were supported by both poly(A) motifs detected by SQANTI3 and *bona fide* TTS (transcription termination sites provided by 3’-seq assays). The 5’ ends of novel transcripts also showed comparable quality to FSM transcripts (Fig. 1E). For example, our long-read ONT CRC transcriptome identified a novel *CDA* transcript derived from an alternative TSS (transcription start site), which is supported by proximal CAGE peaks. We also found *PPIH* transcripts with a novel TTS supported by 3’-seq (Additional file 1: Fig. S3e, f). For some intrinsic sequencing properties, all identified transcripts exhibited an extremely low rate of non-canonical junction usage and reverse transcriptase template switching artifacts (Fig. 1G). Most transcripts (33,271, 58%), particularly novel transcripts, were expressed in only one sample, and 3513 isoforms (6%) were detected in all samples (Additional file 1: Fig. S3g). Notably, rarefaction curve analysis revealed that the discovery of known transcripts reached saturation, yet the discovery of novel transcripts remained unsaturated (Additional file 1: Fig. S3h). Our study provides a high-resolution annotation for the CRC transcriptome.

### Characteristics of novel long-read transcripts

**Fig. 2:**
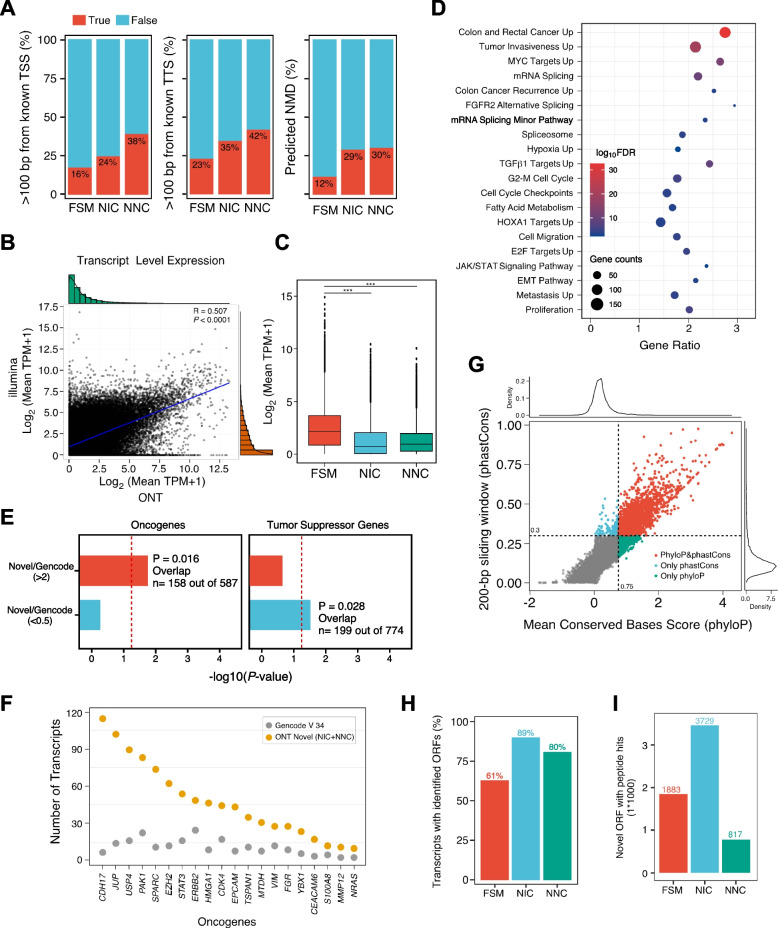
Characteristics of long-read ONT novel transcripts. **A** Percentage of long-read ONT transcripts with TSS 100 bp away from known TSS (left), with TTS 100 bp away from known TTS (center), and with predicted nonsense-mediated mRNA decay (right). **B** Correlation of transcript-level expression calculated by short-read and long-read sequencing. **C** The average expression distribution of different types of transcripts, Wilcoxon signed-rank test, *** *P* < 0.001. **D** Pathways significantly enriched for genes with an elevated number of novel transcripts detected by long-read ONT sequencing in CRC. Bubble size denotes the number of genes with novel transcripts in each pathway, and color denotes the significance. **E** Enrichment analysis of oncogenes and tumor suppressor genes with novel transcripts detected by long-read ONT sequencing in cancer.** F** Number of novel long-read ONT transcripts compared to annotated GENCODE v34 transcripts for selected oncogenes. **G** Correlation between phyloP score and phastCon score of novel transcripts. **H** Percent of long-read isoforms predicted to contain an ORF by Transdecoder.** I** Number of predicted ORFs validated by CPTAC LC–MS/MS proteomics data

Out of the 56,790 novel transcripts discovered, 36,489 (64%) were classified as NNC and 15,640 (27%) were classified as NIC, highlighting the intricate complexity of the CRC transcriptome. Interestingly, we observed a strong positive correlation between the number of exons and the number of novel transcripts, indicating that higher exon complexity leads to an expanded transcript repertoire (Additional file 1: Fig. S4a). Novel transcripts may be derived from new transcriptional start sites (TSSs) and transcriptional terminate sites (TTSs). In fact, novel transcripts with new TSSs were more likely to contain premature stop codons, which are associated with nonsense-mediated mRNA decay (NMD) (Fig. 2A). Furthermore, novel transcripts tended to have more exons but shorter coding sequence. We also observed that NIC transcripts had longer transcripts compared to NNC transcripts (Additional file 1: Fig. S4b-e). The proportion of highly expressed novel transcripts was found to be lower than that of the reference transcripts (Additional file 1: Fig. S4f).

We next compared the genes and transcript expression levels generated by long-read ONT and short-read Illumina platforms. As expected, gene and transcript quantifications from long-read data were highly concordant with those from short-read data (Spearman correlation coefficient *r* = 0.666 for genes and 0.507 for transcripts, respectively; Fig. 2B). Further analyses indicated that the average expression level of novel transcripts (NIC + NNC) was lower than that of known transcripts (FSM) (Fig. 2C). We then conducted pathway enrichment analysis on individual genes with a high gain of novel splice isoforms, selecting those genes with a twofold increase for analysis (see Additional file 2: Table S4). Our results showed that genes with a high gain of novel isoforms are associated with cancer-related signatures, such as RNA splicing, TGF pathway, JAK/STAT pathway, and MYC pathway (Fig. 2D). Notably, oncogenes gained a substantial number of novel transcripts that may play an important role in carcinogenesis and tumor progression. Further analysis indicated that oncogenes were significantly overrepresented in the gene list with a twofold increase, while tumor suppressor genes were underrepresented in this gene set (Fig. 2E). Our analysis revealed that certain oncogenes, including *CDH17*, *EZH2*, and *ERBB2*, exhibited a higher number of novel isoforms (Fig. 2F). Several novel transcript isoforms were validated using qRT-PCR (Additional file 1: Fig. S4g). These findings suggest a potential role for these isoforms in CRC development and progression.

To evaluate the function of the non-coding regions of the genome, which comprise over 98% of the genome and are largely unknown, we assessed the evolutionary conservation of the novel non-coding transcripts. We used phyloP and phastCons scores to perform sequence conservation analysis and found that the novel transcripts had higher conservation scores than random control regions (Fig. 2G). To further characterize the identified novel transcripts that might function as putative protein-coding genes, we evaluated those transcripts with high coding potential. We extracted open reading frames (ORF, length ≥ 300 bp) via sequence homology analysis against the Uniprot database using blastp and functional protein domain prediction using HMMER against the Pfam database [31]. The results indicated that the majority of novel transcripts had the potential to encode proteins (Fig. 2H and Additional file 1: Fig. S5a). To determine whether long-read ONT novel transcripts can encode novel proteins, we compared the predicted ORFs to their closest match in the UniProt database. The results indicated the majority of FSM transcripts encoded ORFs were > 99% identical to the protein in the UniProt database (Additional file 1: Fig. S5b). In contrast, fewer ORFs encoded by NIC or NNC transcripts were annotated in the UniProt database, and a large proportion of ORFs was complete, suggesting that the long-read ONT method may provide much more information for CRC transcriptome.

Beyond transcript annotation, our pipeline leveraged existing CRC proteomics data from the Clinical Proteomic Tumor Analysis Consortium (CPTAC) project to validate our novel ORFs. We intersected our data by spectral matching novel ORFs encoded peptides with 97 publicly available CRC samples and 100 adjacent normal samples profiled by TMT10plex MS/MS proteomics. The results showed that 3729 NIC and 817 NNC novel ORFs encoded peptides were supported by LC–MS/MS data. Additionally, we also found 1883 annotated FSM transcripts producing novel ORFs not present in the UniProt database (Fig. 2I and Additional file 2: Table S5). Of all the ORFs with peptide support, we used the DeepLoc [33], a deep neural network-based tool, to predict the subcellular location of these proteins, with about half located in the cytoplasm or nucleus (Additional file 1: Fig. S5c). The TMHMM analyses showed that 606 novel ORFs had transmembrane domains, suggesting the important role of these previously undefined proteins (Additional file 1: Fig. S5d). Although validating novel transcripts with unique peptides is challenging, LC–MS/MS analysis detected the protein products of three selected novel transcripts (*MSTRG.373437.7*, *MSTRG.17227.18*, and *MSTRG.42052.109*) at their isoform-specific junction regions (Additional file 1: Fig. S5e). These results suggested that previously undetected transcripts can encode novel protein isoforms, which might play a role in producing neoantigens in cancer.

We next systematically analyzed the gene fusion based on the long-read ONT sequencing of CRC samples [27]. The results revealed that a total of 4538 fusion events were detected in our CRC cohort, of which the *ARHGEF3-CNTNAP2*, *A2MP1-PTMA*, and *ACAT2-TCP1* fusion events were considered to be the frequent events in CRC (Additional file 2: Table S6). The majority of identified fusion transcripts have not been detected, which indicates the role of this “dark matter” in CRC, allowing for further investigation.

### The landscape of dysregulated AS events in CRC transcriptome

**Fig. 3:**
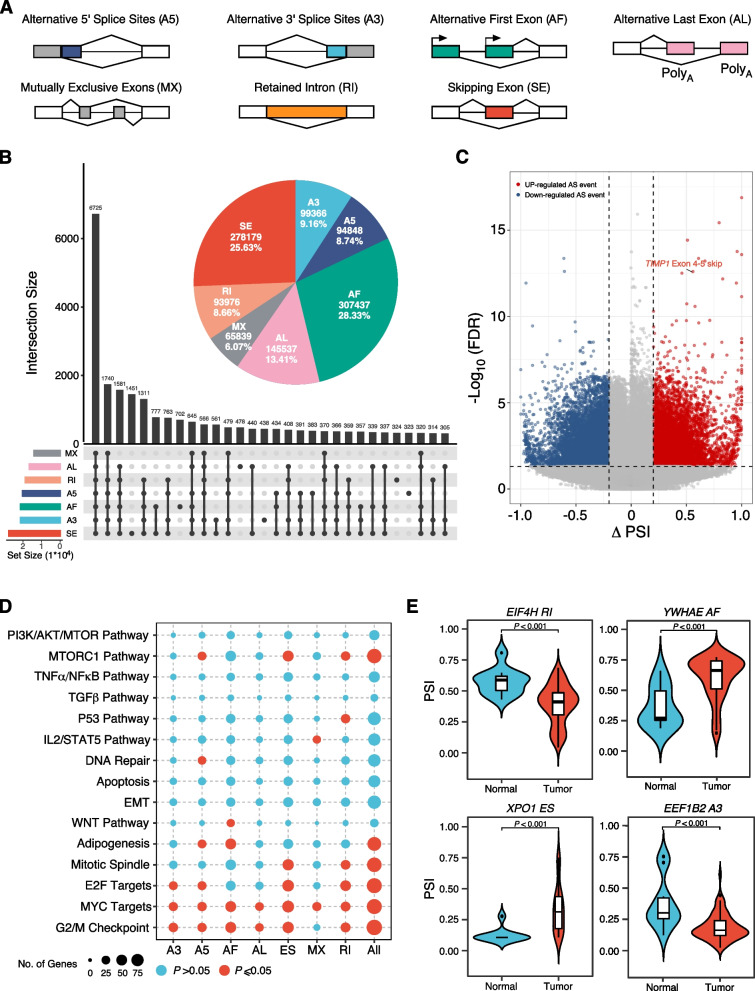
Identification of alternative splicing events in CRC. **A** A schematic diagram of the seven types of AS events including A5: Alternative 5’ Splice Site, A3: Alternative 3’ Splice Site, AF: Alternative First Exon, AL: Alternative Last Exon A3, MX: Mutually Exclusive Exon, RI: Retained Intron, and SE: Skipping Exon. **B** UpSet plot of interactions between the seven types of detected AS events in CRC. Percentage of seven types of AS events detected in CRC (upright). One gene may have up to all seven types of alternative splicing. **C** The volcano plot visualizing DEAS events between CRC and adjacent normal tissues. The red and blue points in the plot represent the DEAS events with statistical significance. **D** Enrichment analysis of genes involved DEAS events for hallmark pathways from MSigDB. Each row represents a hallmark geneset/pathway, whereas each column indicates a type of DEAS events. **E** Distribution of PSI for *EIF4H*, *YWHAE*, *XPO1*, and *EEF1B2* in CRC and adjacent normal tissues. *P values* were calculated by two-sided Mann–Whitney test

We annotated the AS events based on ONT long-read data using SUPPA2 software [38] and quantified the levels of seven types of splicing events, including skipping exons (SE), alternative 3’ sites (A3), alternative 5’ sites (A5), alternative first exon (AF), alternative last exon (AL), intron retention (RI), and mutually exclusive exon (MX) categories (Fig. 3A). We identified a total of 2,363,976 AS events, with AF events contributing to the majority. Of these, only 565,952 AS events were identified using GENCODE v34 reference (Additional file 1: Fig. S6a). To obtain a reliable AS events dataset, we implemented rigorous filtration criteria (i.e., PSI frequency ≥ 75% and average PSI value ≥ 0.05). As a result, we obtained a total of 1,085,182 AS events, of which 352,238 AS events were identified based on the GENCODE v34 reference (Additional file 1: Fig. S6b, c). The UpSet plot revealed that 6725 genes have all seven types of AS events. It is also noteworthy that > 90% of genes have two or more AS events (Fig. 3B). The various combinations of these AS events largely enriched the diversity of the CRC transcriptome.

To explore the aberrant AS events that occur in CRC. Differential analysis was performed on AS events between tumor and adjacent normal samples, resulting in the identification of 25,002 differentially expressed AS (DEAS) events with an adjusted *P*-value ≤ 0.05 and |ΔPSI|≥ 0.2 (Fig. 3C and Additional file 2: Table S7). The majority of DEAS events were AF events (8898, 35.59%) (Additional file 1: Fig. S7a). There was no significant difference in the distribution of ΔPSI across seven types of AS events (Additional file 1: Fig. S7b). Unsupervised hierarchical clustering based on PSI values of DEAS events was able to distinctly separate tumor and adjacent normal samples (Additional file 1: Fig. S7c). Next, we conducted gene set enrichment analysis (GSEA) on the parental genes that harbored DEAS events to determine their association with hallmark pathways. The GSEA results showed that genes with DEAS events were enriched in pathways such as G2/M cell cycle checkpoint, E2F signaling pathway, MYC signaling pathway, Mitotic Spindle, and MTORC1 signaling pathway (Fig. 3D). Intriguingly, different types of AS events were significantly enriched in specific pathways. For example, only differentially expressed AF events were overrepresented in the WNT signaling pathway, while the p53 signaling pathway was exceptionally enriched in RI events (Fig. 3D). Furthermore, we identified specific genes involved in MYC signaling pathways that underwent significant AS changes during tumorigenesis. For instance, *EIF4H* displayed loss of intron retention that was highly specific to CRC. *YWHAE* showed significant changes in alternative first exon usage between tumor and adjacent normal samples. *XPO1* exhibited significant exon skip alterations in tumors compared to adjacent normal samples, while the alternative 3’ change of *EEF1B2* showed a significant difference between tumor and adjacent normal samples (Fig. 3E). These findings suggest that DEAS events might act as complicated roles through interacting with multiple cancer-related pathways in CRC.

Splicing factors (SFs) have been documented to play pivotal roles in RNA splicing process, and a substantial portion of DEAS events was directly regulated by the differentially expressed SFs [46]. Among those previously curated 67 SF genes, we found that 17 SF genes were differentially expressed between tumor and adjacent normal samples (Additional file 2: Table S8). We then constructed dysregulation networks by calculating the correlation between expression levels of differentially expressed SFs and DEAS events (Additional file 1: Fig. S8a and Additional file 2: Table S9). In the dysregulation network, we found that 13 SFs (11 upregulated and 2 downregulated SFs in tumors) orchestrated thousands of DEAS events involved in various biological pathways. Notably, *ELVAL3* and *QKI* were observed to modulate a greater number of DEAS events and pathways, indicating that they may play crucial roles in CRC (Additional file 1: Fig. S8b). The pathway enrichment analysis on the DEAS events regulated by SFs indicated that most pathways were significantly associated with cancer metabolisms, such as hypoxia, HIF1A signaling pathway, and Ribosome (Additional file 1: Fig. S8c). Overall, our findings demonstrate that SFs regulate both metabolism-related and other crucial pathways in CRC. In particular, SFs such as *ELVAL3*, *QKI*, *RBM5*, *HNRNPA1*, and *SF1*, which are at the center of the regulatory dysregulation network, may play pivotal roles in CRC carcinogenesis and progression.

### Clinical outcome associated with alternative splicing events

**Fig. 4:**
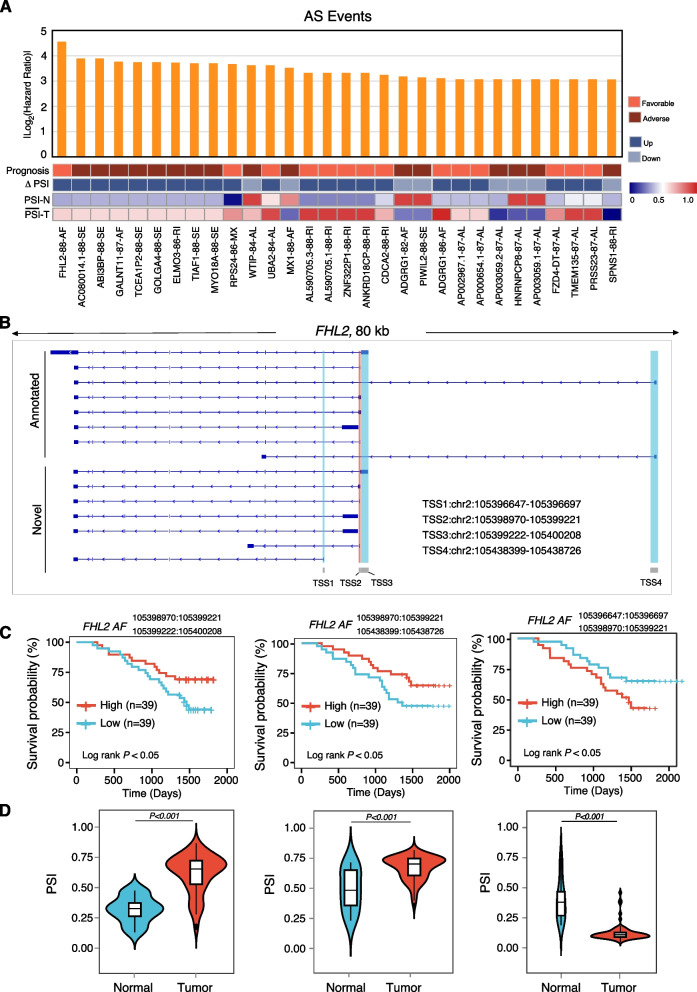
Identifications of prognostic DEAS events in CRC. **A** Top 30 prognostic DEAS events are shown, ranked by differential survival. AS events are labeled with gene name, AS event type, and the number of patients. Each AS event is depicted in the heatmap, including survival prognosis, differential status, and average PSI values. **B** Gene structure of the known and novel TSS of *FHL2* transcript according to the ONT long-read transcriptome of CRC tissues. **C** Kaplan–Meier survival analysis of the correlation between three types of FHL2 DEAS events and patients’ overall survival rate, log-rank test. **D** Distribution of PSI for 3 types of *FHL2* AF events in CRC and adjacent normal tissues, *P values* were calculated by Two-sided Mann–Whitney test

Due to the highly variable features of AS events, we next explored whether their usage might highlight new prognostic biomarkers in CRC. We performed univariate Cox analysis to assess the correlation between the overall survival rate and each DEAS event. In total, we have identified 1472 DEAS events corresponding to 1241 genes that showed prognostic significance (Additional file 2: Table S10). Of these, 809 DEAS events were associated with a favorable prognosis, while 663 DEAS events were associated with a worse prognosis. Notably, we found 38 genes were identified having both favorable and worse prognostic AS events. The top 30 DEAS events corresponding to 27 distinct genes correlated with overall survival in CRC were shown (Fig. 4A). Intriguingly, four AS events of *FHL* were associated with overall survival. The skipped exon and alternative first exon of *FHL2* were associated with a decreased survival time, while the other two alternative first exon events of *FHL2* were associated with prolonged overall survival time in CRC patients (Fig. 4B–D). Overall, our findings suggest that DEAS events may serve as valuable prognostic biomarkers for CRC. The complexity of DEAS events in genes such as *FHL2* may lead to specific isoforms during tumorigenesis and progression, highlighting the need for further research to elucidate the functional roles of different isoforms. Our study provides new insights into the close relationship between DEAS events and CRC prognosis, offering a promising avenue for future investigation.

To further investigate the dysregulation of alternative splicing in CRC. We analyzed the DEAS events between the advanced stage (AJCC Stage III + IV) and early-stage (AJCC Stage I + II) CRC patients. In total, we identified 998 AS events to be differentially expressed (adjusted *P*-value ≤ 0.05 and |ΔPSI|≥ 0.2, Additional file 2: Table S11). Furthermore, we analyzed the metastasis-related DEAS, and the result showed that 596 DEAS events were identified to be upregulated in CRC samples with distant metastasis, while 583 DEAS events were found to be significantly decreased (Additional file 2: Table S12). The microsatellite instability (MSI) was considered to be a robust prognostic and predictive biomarker to guide therapeutic decision-making including the use of immunotherapy [47]. We predicted the CRC MSI status based on the transcriptome data by PreMSIm [44], and the results indicated that 20 patients were predicted as MSS/MSI-L status, while the 58 remaining CRC patients were predicted to be MSI-H. Further analysis showed that 744 DEAS events were identified to be upregulated in CRC samples with MSI-H, while 633 DEAS events were identified to be significantly decreased in MSI-H patients (Additional file 2: Table S13), implying that alternative splicing dysregulation may be associated with immunotherapy response. These results suggest that dysregulation of alternative splicing is closely related to CRC progression and therapeutic choice, indicating that it may serve as important biomarkers and potential therapeutic targets.

Besides clinicopathological correlation, we also investigate the relationship between the molecular subtypes and alternative splicing. The consensus molecular subtypes (CMS) is a robust and useful classification system that allows to categorize CRC patients into four subtypes by transcriptome analysis [48]. The CMS classification system is significantly correlated with CRC patient’s overall survival rate and can guide therapeutic decision-making in clinical practice. Firstly, we predicted the CMS for each patient based on the transcriptome data by CMScaller [43]. Then, we analyzed the CMS specific AS events in different CMS subtypes. Each CMS subtype-specific AS event was defined as high level of PSI value in the subtype versus the remaining subtypes (adjusted *P*-value ≤ 0.05 and |ΔPSI|≥ 0.2). The results showed that a total of 4823 AS events were considered to be CMS subtype specific, of which 1579, 930, 1324, and 990 AS events were found to be highly prevalent in CMS1, CMS2, CMS3, and CMS4 CRC patients, respectively (Additional file 2: Table S14). Regarding CMS subtypes, it is well appreciated that tumor microenvironment including stromal or immunoinfiltration largely affected the CRC prognosis [49]. To analyze the correlation between tumor microenvironment and alternative splicing, we employed the estimate to characterize the stromal and immune cell infiltration [45], and then analyzed the correlation between CRC microenvironment and splicing alterations in CRC samples. The results showed that 237 AS events were found to be significantly correlated with stromal content, while 100 AS events were found to be significantly correlated with immunoinfiltration (adjusted *P*-value ≤ 0.05 and |Spearman correlation coefficient *r*|≥ 0.5), suggesting the potential role of alternative splicing in CRC microenvironment remodeling (Additional file 2: Table S15 and Additional file 2: Table S16).

### Dysregulation of TIMP1 exon 4–5 splicing in CRC

**Fig. 5:**
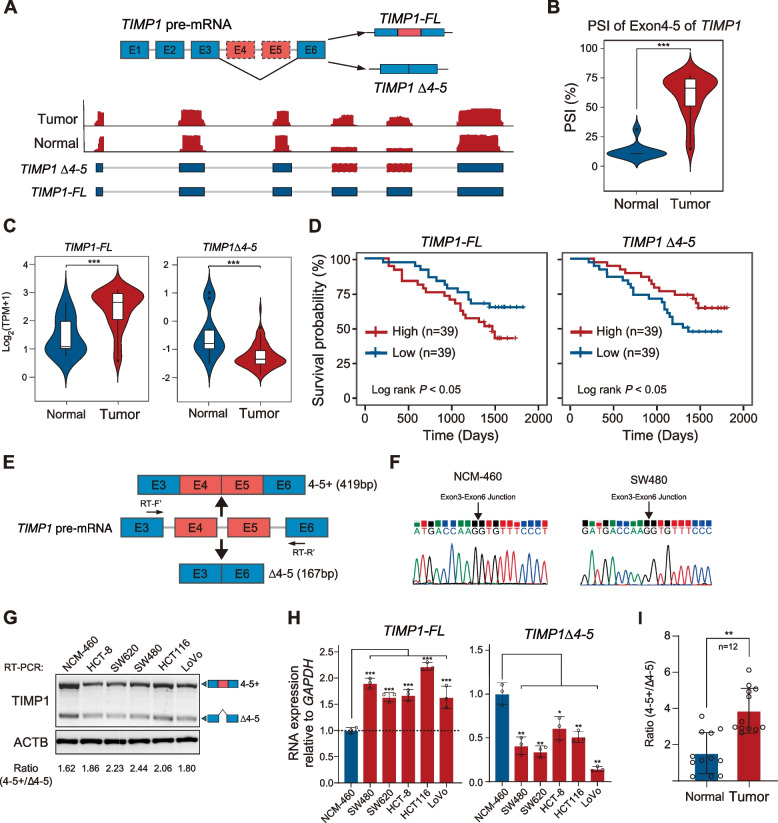
Dysregulation of TIMP1 exon 4–5 splicing in CRC.** A** Genome browser view depicts the coverage of exons in TIMP1 in representative normal and tumor tissues. **B** Distribution of PSI value of *TIMP1* exon 4–5 skip events in CRC and adjacent normal tissues, *P values* were calculated by two-sided Mann–Whitney test, *** *P* < 0.0001. **C** Expression level of full-length TIMP1 transcript (*TIMP1-FL*, left) or TIMP1 transcript with exon 4 and 5 exclusion (*TIMP1 Δ4-5*, right) in CRC and adjacent normal tissues, *P values* were calculated by two-sided Mann–Whitney test, *** *P* < 0.001. **D** Kaplan–Meier survival analysis of the correlation between *TIMP1-FL* (left) or *TIMP1 Δ4-5* (right) expression and patients’ overall survival rate, log-rank test. **E** The Scheme describes the primer design strategy to characterize the exon 4 and 5 inclusion or exclusion of TIMP1. **F** Sanger sequencing analysis indicated the exon3-exon6 junction in NCM-460 and SW480 cells. **G** RT-PCR results of TIMP1 in different CRC cancer cell lines and normal human colon mucosal epithelial cell line. **H** qRT-PCR results of *TIMP1-FL* (left) or *TIMP1 Δ4-5* (right) expression in CRC cancer cell lines and normal human colon mucosal epithelial cell line. *P values* were calculated by two-sided Student’s *t* test, * *P* < 0.05, ** *P* < 0.01, *** *P* < 0.001. **I** qRT-PCR results depict the ratio of transcripts with or without exon 4–5 in 12 pairs of CRC and adjacent normal tissues. *P values* were calculated by two-sided Student’s *t* test, ** *P* < 0.01

Among those identified DEAS events, the PSI value of TIMP1 exon 4–5 was significantly elevated in CRC samples (Fig. 3C). The human *TIMP1* gene spans approximately 4.4 kb and contains 6 exons. In this study, we found for the first time that *TIMP1* gene can generate a new transcript by alternative exon skipping exon 4–5 (*TIMP1 Δ4-5*) based on our ONT long-read data (Fig. 5A). The PSI value of *TIMP1 Δ4-5* was significantly elevated in CRC samples (Fig. 5B). To investigate whether *TIMP1* isoforms with (*TIMP1-FL*) or without of exon 4–5 (*TIMP1 Δ4-5*) is differentially expressed between CRC and adjacent normal tissues, we analyzed their expression values using Illumina short reads data. The results showed that the mRNA expression level of *TIMP1-FL* was significantly upregulated in cancerous tissues, while the expression level of *TIMP1 Δ4-5* was significantly decreased in CRC tissues (Fig. 5C, D). Moreover, the increased expression of *TIMP1-FL* was significantly associated with poorer overall survival, while the increased expression of *TIMP1 Δ4-5* leads to better survival in CRC patients (Fig. 5C, D and Additional file 1: Fig. S9a).

To further validate the splicing role of *TIMP1* gene in CRC, we designed three sets of primers. Primer set 1 covered a fragment spanning exon 3 to exon 6 to validate *TIMP1-FL* and *TIMP1 Δ4-5* transcripts (Fig. 5E). Moreover, primer set 2, which covered the exon 3/exon 4 and exon 5/exon 6 junctions, was designed to quantify *TIMP1-FL* transcript. Primer set 3 covered the exon 3/exon 6 junction and was used to quantify *TIMP1 Δ4-5* transcript (Additional file 1: Fig. S9b). Sanger sequencing results confirmed the presence of *TIMP1 Δ4-5* isoform in both NCM-460 and SW480 cells (Fig. 5F). Further RT-PCR and qRT-PCR results showed that the expression level of *TIMP1-FL* was significantly upregulated, while the expression level of *TIMP1 Δ4-5* was significantly downregulated in CRC cell lines (Fig. 5G, H). Importantly, we identified the same expression pattern in clinical CRC samples (Fig. 5I and Additional file 1: Fig. S9c). The ratio of exon 4–5 inclusion versus exclusion (*TIMP1-FL*/*TIMP1 Δ4-5* mRNA) was dramatically increased in CRC tissues compared with adjacent normal tissues. The data suggested that dysregulation of *TIMP1* exon 4–5 alternative splicing exists in CRC samples.

#### TIMP1 Δ4-5 and TIMP-FL have antithetical functions in CRC carcinogenesis

**Fig. 6:**
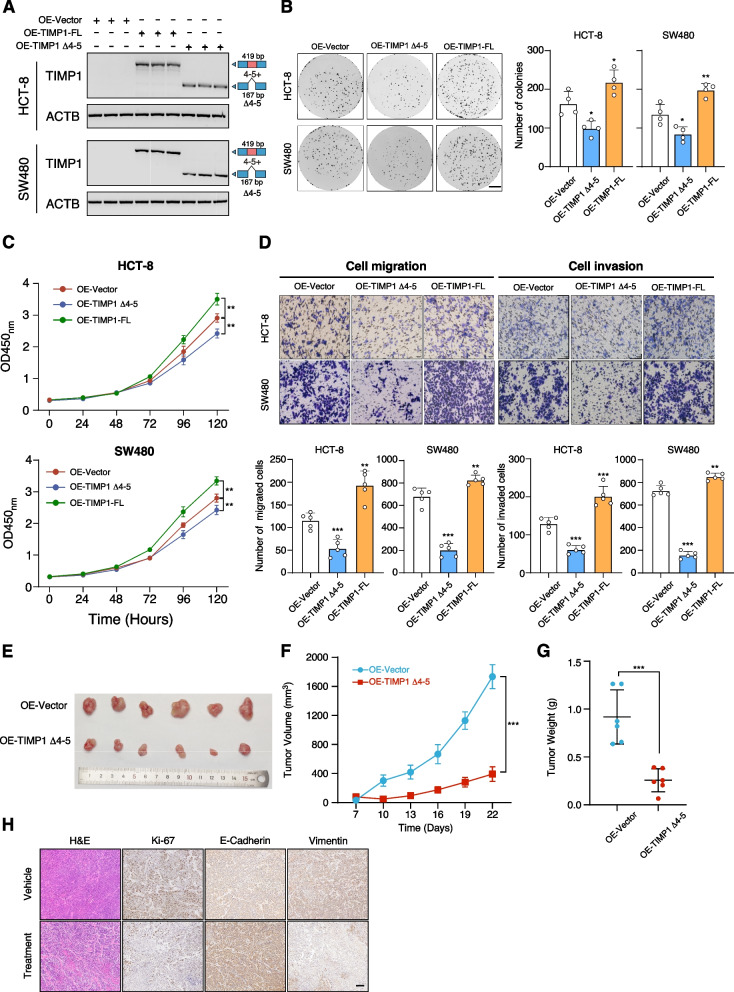
TIMP1 *Δ*4-5 and TIMP-FL have antithetical functions in CRC carcinogenesis.** A** Exogenous overexpression of TIMP1 *Δ*4-5 and TIMP-FL in TIMP1-KD HCT-8 and SW480 cells. **B, C** Colony formation (**B**) and MTT (**C**) assay of exogenous overexpression of TIMP1 *Δ*4-5 and TIMP-FL in TIMP1-KO HCT-8 and SW480 cells. *P* values were calculated by two-sided Student’s *t* test, * *P* < 0.05, ** *P* < 0.01. **D** Tumor cell migration and invasion assay of exogenous overexpression of TIMP1 *Δ*4-5 and TIMP-FL in TIMP1-KO HCT-8 and SW480 cells. *P* values were calculated by two-sided Student’s *t* test, ** *P* < 0.01, *** *P* < 0.001. The migrated or invaded cells were quantified by counting in five fields. Scale bar, 100 μm. **E** Xenograft mouse model established using SW480 cells was stably infected with lentivirus-based TIMP1 *Δ*4-5 or empty vector in BALB/c nude mice (*n* = 6 mice per group). In vivo generated tumors are depicted. **F, G** Analysis of tumor growth (**F**) and weight (**G**) in the xenograft mouse model. Data are presented as mean ± SEM of *n* = 6 mice per group. Two-way ANOVA and one-way ANOVA followed by Tukey test. **H** Representative H&E and IHC images of randomly selected tumors were shown. Scale bar, 100 µm

It has been documented that *TIMP1-FL* expression is correlated with tumorigenesis and metastasis of human CRC [23]. To investigate the individual contributions of *TIMP1 Δ4-5* and *TIMP1-FL* in CRC, we overexpressed *TIMP1 Δ4-5* and *TIMP1-FL* to CRC and we overexpressed *TIMP1 Δ4-5* and *TIMP1-FL* in CRC cell lines. The overexpression efficiencies were confirmed by qRT-PCR (Additional file 1: Fig. S9d). Our findings showed that overexpression of *TIMP1 Δ4-5* significantly suppressed cell growth, migration, and invasion of SW480 and HCT-8 cells, while overexpression of *TIMP1-FL* remarkably increased cell growth, migration, and invasion abilities of both SW480 and HCT-8 cell lines reduced cell migration and invasion abilities, which is in concordance with previous study (Fig. 6A–D) [23]. Conversely, we employed siRNA targeting exon 3/exon 6 junction to knockdown *TIMP1 Δ4-5* expression in CRC cells. The results indicated that knockdown of *TIMP1 Δ4-5* had no effects on *TIMP1-FL* expression. Knockdown of *TIMP1 Δ4-5* significantly increased cell proliferation, migration, and invasion abilities (Additional file 1: Fig. S9e-g). The data suggested that dysregulation of *TIMP1 Δ4-5* may function as a suppressor of CRC carcinogenesis.

We also evaluated the in vivo effects of *TIMP1 Δ4-5* overexpression on tumor growth in a nude mouse xenograft model, which showed that cells overexpressing *TIMP1 Δ4-5* generated smaller tumors compared to controls (Fig. 6E–H). Collectively, our data indicate that *TIMP1-FL* and *TIMP1 Δ4-5* play distinct roles in CRC carcinogenesis and that *TIMP1 Δ4-5* may act as a potential tumor suppressor in CRC.

### SRSF1 sustains the exon 4–5 inclusion of TIMP1 in CRC

**Fig. 7:**
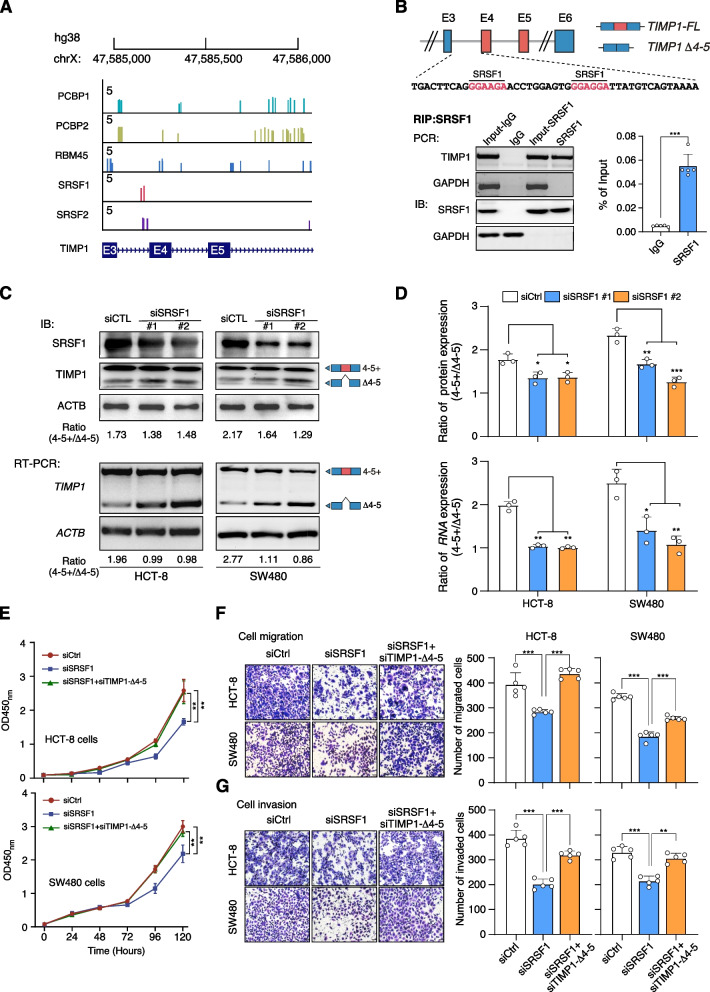
SRSF1 sustains the exon 4–5 inclusion of TIMP1 in CRC.** A** RBPMap analysis shows the potential binding of several RNA-binding proteins in *TIMP1* pre-mRNA. **B** RNA immunoprecipitation (RIP)-qPCR analysis of SRSF1 binding with *TIMP1* pre-mRNA in SW480 cells. Data were shown as mean ± SD. *P* values were calculated by two-sided Student’s *t* test, *** *P* < 0.001. **C** Western blot and RT-PCR analysis of TIMP1 exon 4 and 5 skip after transfecting with si-SRSF1 or control siRNA in HCT-8 and SW480 cells. **D** Quantitative analysis of the western blot and RT-PCR results in **C** by the ImageJ software. Data were shown as mean ± SD. *P* values were calculated by two-sided Student’s *t* test, ** *P* < 0.01, *** *P* < 0.001. **E** MTT assay of HCT-8 and SW480 cells transfected with si-SRSF1, si-SRSF1 + si-TIMP1 *Δ*4-5, and control siRNA. Data were shown as mean ± SD. *P* values were calculated by two-sided Student’s *t* test, ** *P* < 0.01. **F, G** Tumor cell migration (**F**) and invasion (**G**) assay of HCT-8 and SW480 cells with indicated treatments. *P* values were calculated by two-sided Student’s *t* test, ** *P* < 0.01, *** *P* < 0.001. The migrated or invaded cells were quantified by counting in five fields. Scale bar, 100 μm

To better understand how splicing factors regulate TIMP1 exon 4–5 inclusion, we used RBPmap (http://rbpmap.technion.ac.il/) to predict potential splicing factors that bind to *TIMP1* pre-mRNA [50]. The result showed that *TIMP1* exon 4–5 inclusion or exclusion is regulated by five splicing factors potentially (*SRSF1*, *PCBP1*, *PCBP2*, *RBM45*, and *SRSF2*, Fig. 7A). To confirm these findings, we individually silenced each of these splicing factors in CRC cells and performed RT-PCR to detect *TIMP1* exon 4–5 inclusion. We found that knockdown of *SRSF1* significantly increased exon 4–5 inclusion (Additional file 1: Fig. S10a). We then employed an anti-SRSF1 antibody to perform RIP assay in SW480 cell to determine whether SRSF1 directly binds to *TIMP1* mRNA. The results showed that anti-SRSF1, but not IgG, directly binds to *TIMP1* mRNA (Fig. 7B). We further silenced *SRSF1* expression in SW480 and HCT-8 cells to investigate the expression change of *TIMP1-FL* and *TIMP1 Δ4-5*. Our findings showed that SRSF1 knockdown significantly attenuated *TIMP1-FL* expression but increased *TIMP1 Δ4-5* expression compared to Scram knockdown cells, while total *TIMP1* level remained unchanged (Additional file 1: Fig. S10c, d). Further analyses showed that *SRSF1* knockdown remarkably attenuated the *TIMP1-FL/TIMP1- Δ4-5* expression ratio at both RNA and protein level (Fig. 7C, D). Overall, these results suggest that *SRSF1* plays an important role in regulating *TIMP1* exon 4–5 inclusion and exclusion in CRC.

*SRSF1* has been implicated in cancer progression [51, 52]. We herein investigated the expression of *SRSF1* in CRC based on our sequencing data. Our analysis revealed that *SRSF1* was significantly upregulated in CRC compared with adjacent normal tissues (Additional file 1: Fig. S10b), indicating its potential as a driver of CRC carcinogenesis. We silenced *SRSF1* expression in SW480 and HCT-8 cells and observed a significant inhibition of cell growth, which was rescued by further knockdown of *TIMP1 Δ4-5* expression (Fig. 7E). Moreover, transwell assays indicated that knockdown of SRSF1 markedly reduced the cell migration and invasion abilities, which were further promoted by knockdown of *TIMP1 Δ4-5* (Fig. 7F, G). These results suggest that *SRSF1* promotes CRC carcinogenesis via promoting the exon 4–5 inclusion of *TIMP1* gene.

### Therapeutic strategy by inducing TIMP1 exon 4–5 exclusion using CRISPR/dCasRx system

**Fig. 8:**
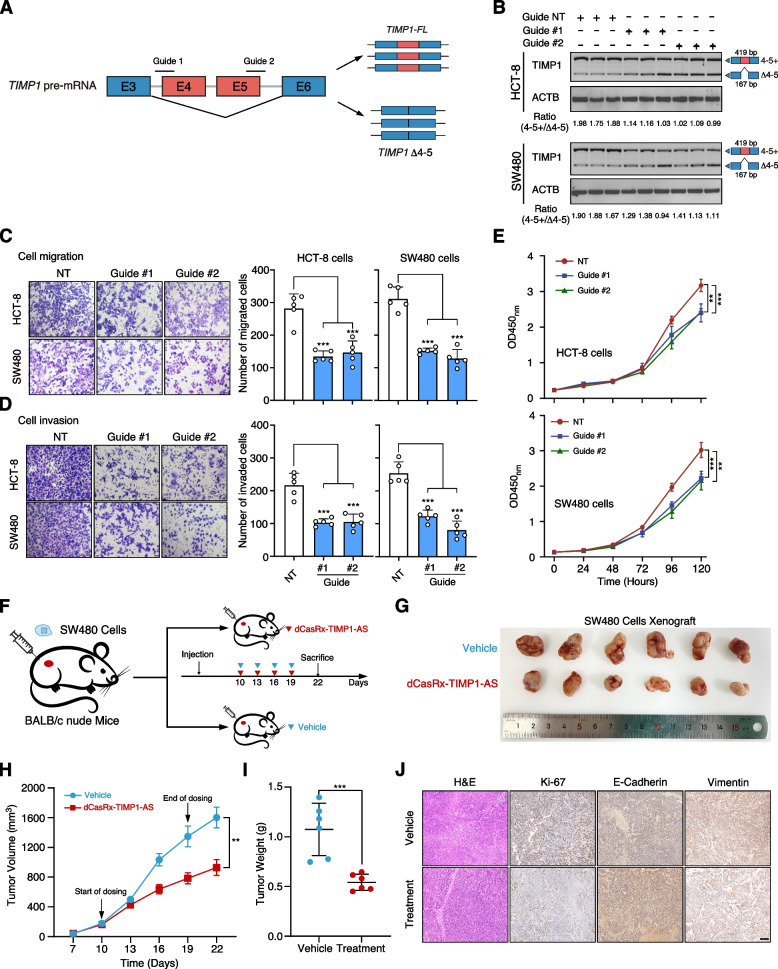
Therapeutic value of targeting TIMP1 alternative splicing in CRC.** A** The scheme depicts targeting the TIMP1 alternative splicing by CRISPR/dCasRx system. **B** RT-PCR results show that CRISPR/dCasRx targeting significantly decrease the ratio of TIMP1 transcripts with or without exon 4–5 in HCT-8 and SW480 cells transfecting with guides targeting intron–exon boundary compared with non-target guide. The ratio was quantified with ImageJ software. **C, D** Tumor cell migration (**C**) and invasion (**D**) assay of HCT-8 and SW480 cells with indicated treatments. *P* values were calculated by two-sided Student’s *t* test, *** *P* < 0.001. The migrated or invaded cells were quantified by counting in five fields. Scale bar, 100 μm. **E** MTT assay of HCT-8 and SW480 cells with indicated treatments. Data were shown as mean ± SD. *P* values were calculated by two-sided Student’s *t* test, ** *P* < 0.01, *** *P* < 0.001. **F** The injection schematic for in vivo anti-tumor effect analysis of targeting TIMP1 exon 4–5 skip by CRISPR/dCasRx system. **G** Xenograft mouse model established using SW480 cells in BALB/c nude mice with indicated treatment (*n* = 6 mice per group). In vivo generated tumors are depicted. **H, I** Analysis of tumor growth (**H**) and weight (**I**) in the xenograft mouse model. Data are presented as mean ± SEM of *n* = 6 mice per group. Two-way ANOVA and one-way ANOVA followed by Tukey test. **J** Representative H&E and IHC images of randomly selected tumors are shown. Scale bar, 100 µm

In this study, we utilized the CRISPR/dCasRx system to manipulate the alternative splicing of *TIMP1* gene. Previous studies have shown that targeting the regulatory splicing site flanking the alternatively spliced exon with dCasRx can induce exon skipping [24]. To evaluate the position-dependent effect of different gRNAs, we designed six gRNAs mapping to different loci of *TIMP1* pre-mRNA genomic location (Additional file 1: Fig. S11). We transfected dCasRx and each gRNA into SW480 cells and used RT-PCR to evaluate *TIMP1* exon 4–5 exclusion after 48 h. The results showed gRNA#1 and gRNA#2 that located in donor and acceptor sites significantly reduced the relative *TIMP1-FL*/*TIMP1 Δ4-5* ratio by nearly 50% relative to a non-target control (Fig. 8A). These results demonstrate that the CRISPR/dCasRx system was able to efficiently induce the exon 4–5 exclusion of *TIMP1* in the in vitro cultured CRC cells.

We then investigated the potential of the CRISPR/dCasRx system in targeting *TIMP1* alternative splicing. We employed transwell assay after transfecting dCasRx and gRNA to induce *TIMP1* exon 4–5 exclusion. The results indicated that inducing *TIMP1* exon 4–5 exclusion significantly inhibited cell migration and invasion abilities of SW480 and HCT-8 cells compared to the non-target control (Fig. 8B–D). Additionally, cell proliferation was dramatically inhibited upon dCasRx-mediated exon exclusion (Fig. 8E). We further tested this CRISPR/dCasRx-based strategy in vivo by administering subcutaneous injections of SW480 cells to the nude mice. After 1 week post-inoculation, mice received four intratumoral injections of either the TIMP1 CRISPR/dCasRx (dCasRx-TIMP1) vector plus delivery buffer or only the delivery buffer (Fig. 8F). Mice receiving the control delivery buffer exhibited relatively larger neoplasms, while those receiving the dCasRx-*TIMP1* vector treatment showed smaller neoplasms (Fig. 8G-J). CRISPR/dCasRx-based strategy designed to induce *TIMP1* exon 4–5 exclusion may have the potential to restrain the growth of CRC.

## Discussion

Aberrant AS has been implicated as a hallmark of cancer development. However, the RNA splicing landscape of CRC has still not been fully explored [53, 54]. The identification and functional establishment of global AS events poses a formidable challenge. Recently developed long-read sequencing technologies are changing this situation [17, 18, 20, 21]. Several pioneering studies have reported unexpected transcriptome complexity using long-read sequencing technology [20, 55, 56]. In this work, we have generated long-read and short-read RNA-seq data to describe the AS landscape in human CRC. Long-read RNA-seq accurately detected full-length isoforms from start to end, revealing a rich diversity of spliced isoforms. Our findings highlighted the power of novel long-read sequencing approaches for transcriptional profiling.

To the best of our knowledge, our work represents the most comprehensive characterization of full-length transcripts and splicing isoform diversity in CRC yet undertaken. We identified a large number of novel transcripts that have never been annotated, which largely expanded the transcriptomic complexity. Notably, we detected novel full-length transcripts from several previously annotated genes, such as *CTNNB1* and *EZH2*. Moreover, we demonstrated that oncogenes and tumor suppressor genes exhibited significantly different RNA isoform diversity in CRC samples, unveiling novel transcripts that may play important roles in carcinogenesis. By generating sequencing reads spanning the entire transcriptome, it is possible to systematically characterize the expressed RNA isoforms repertoire and comprehensively evaluate the prevalence of AS. Even due to the long-time storage of CRC samples, the RNA may suffer from slight degradation which leads to the relatively low fraction of full-length mitochondrial transcripts detected by ONT. We also investigate the coding potential of previously undetected transcripts by integrating the ONT data with proteomics data. The MS/MS data of CRC were obtained from CPTAC database, and most of the peptides have relatively small length (< 10 AAs), which is difficult to discriminate the protein from novel transcripts or known reference. Further methods may be used to improve the accuracy of novel coding transcripts identification, such as ribosome seq or RNC-seq. Our data confirms the importance of aberrant AS in CRC and highlights its role as an important mechanism underpinning gene regulation in human cancers.

Several findings are particularly striking. First, we discovered that the current gene annotations are still far from complete and the novel transcripts are likely to exist for a large proportion of expressed genes. For example, *CDH17* exhibits a tenfold increase in novel isoforms compared to those currently annotated in existing databases. It has been shown that such incomplete annotation has a great influence on our understanding of tumorigenesis and metastasis. Our resource enhanced our understanding of CRC expressed transcripts repertoire. Second, we were able to highlight the extent to which AS events make a major contribution to isoform diversity in CRC. In particular, we show that AF is a relatively more prevalent form of AS type in CRC samples, which is contrary to previous findings based on short-read RNA-seq data [57]. Third, we highlighted the survival-related AS events based on the prognosis information of CRC patients. These AS events might serve as prognostic markers in facilitating patient outcome estimation.

In vitro and in vivo assays showed that overexpression of *TIMP1 Δ4-5* significantly inhibits CRC cell growth and metastasis. Our further investigation suggests that *SRSF1* promotes CRC carcinogenesis and progression by retaining the exon 4–5 of *TIMP1*. *TIMP1* is frequently overexpressed in a wide range of cancer types, but it remains unclear whether aberrant alternative splicing of TIMP1 is involved in other cancers.

Our long-read RNA-seq data provided the first direct evidence that *TIMP1* is alternative spliced at exon 4–5, demonstrating that *TIMP1* exon 4–5 inclusion is a frequent event in CRC. Here, we found that the *TIMP1-FL* isoform, which includes exon 4–5, is upregulated in CRC tissues, while the *TIMP1 Δ4-5* isoform, which lacks exon 4–5, is downregulated, suggesting an important role in CRC tumorigenesis. Further in vitro and in vivo assays show that overexpression of *TIMP1 Δ4-5* significantly inhibits CRC cell growth and metastasis. Further investigation indicates *SRSF1* promotes CRC carcinogenesis and progression by retaining the exon 4–5 of *TIMP1*. Studies on human cancers indicated that *TIMP1* is frequently overexpressed in a wide range of cancerous types [58]. It remains unclear whether aberrant AS of *TIMP1* is also involved in other cancer types.

Our study suggests that modulating *TIMP1* splicing could be a promising therapeutic strategy for CRC. One promising approach to specifically manipulate splicing at specific site is splice-switching antisense oligonucleotides (SSO). However, SSO is expensive and difficult to set up, which also requires transfecting substantial modified oligonucleotides for an efficient splicing effect, reducing the feasibility in large-scale studies. Recently, it has been reported that catalytic dead mutant Cas13 family member, dCasRx, has shown promise for efficiently mediating alternative splicing patterns in a physiological context. By targeting regulatory splicing sites flanking the alternatively spliced exons, dCasRx can induce exon skipping or increased inclusion by most likely interfering with the recruitment of splicing factors. In our study, a CRISPR/CasRx-based therapeutic strategy to induce the *TIMP1* exon 4–5 exclusion significantly inhibited neoplasm growth in BALB/c nude mice. Our findings demonstrate the potential of dCasRx as an efficient tool for editing RNA splicing and may be broadly applicable as a novel therapeutic strategy for cancer.

## Conclusions

In summary, we have surveyed the landscape of CRC transcriptome using long-read sequencing method. Having observed substantial level AS events and previously unknown transcript isoforms, our results suggested that full-length transcriptome profiling reveals an underexplored area of research which can help the discovery of biomarkers and new drug targets.

### Supplementary Information

**Additional file 1:** **Fig. S1.** Analysis pipeline for long-read isoform annotation, quality control and prediction of protein features. **Fig. S2**. Performance statistics for ONT RNA sequencing. **Fig. S3.** Characteristics of isoforms detected by ONT long-read sequencing. **Fig. S4.** Characteristics of different types of Long read ONT identified transcripts. **Fig. S5.** Novel transcripts detected in colon tumors by ONT long-read sequencing are predicted to impact protein sequence, domains, or localization. **Fig. S6. **Identification of alternative splicing events in CRC. **Fig. S7.** Identification of differentially expressed alternative splicing events in CRC. **Fig. S8. **The regulation network of DEAS events by differentially expressed SFs. **Fig. S9.** Dysregulation of TIMP1 exon 4-5 splicing in CRC. **Fig. S10.** SRSF1 sustains the exon 4-5 inclusion of TIMP1 in CRC. **Fig. S11.** Optimization of guides for targeting the TIMP1 alternative splicing by CRISPR/dCasRx system.

**Additional file 2:** **Table S1-Table S17.**

## Data Availability

The ONT and Illumina sequencing data have been deposited in the NCBI Sequencing Read Archive database (SRA, http://www.ncbi.nlm.nih.gov/sra/) under the accession number SRP303779 (https://trace.ncbi.nlm.nih.gov/Traces/?view=study&acc=SRP303779) and SRP303082 (https://trace.ncbi.nlm.nih.gov/Traces/?view=study&acc=SRP303082), respectively.
