# Supplementary material for: Long-read sequencing reveals the landscape of aberrant alternative splicing and novel therapeutic target in colorectal cancer
Source: Genome Med. 2023 Sep 21;15:76. doi: 10.1186/s13073-023-01226-y (PMC10512518; doi:10.1186/s13073-023-01226-y)
Supplement: Supplementary file 1 — Additional file 1: Fig. S1. Analysis pipeline for long-read isoform annotation, quality control and prediction of protein features. Fig. S2. Performance statistics for ONT RNA sequencing. Fig. S3. Characteristics of isoforms detected by ONT long-read sequencing. Fig. S4. Characteristics of different types of Long read ONT identified transcripts. Fig. S5. Novel transcripts detected in colon tumors by ONT long-read sequencing are predicted to impact protein sequence, domains, or localization. Fig. S6. Identification of alternative splicing events in CRC. Fig. S7. Identification of differentially expressed alternative splicing events in CRC. Fig. S8. The regulation network of DEAS events by differentially expressed SFs. Fig. S9. Dysregulation of TIMP1 exon 4-5 splicing in CRC. Fig. S10. SRSF1 sustains the exon 4-5 inclusion of TIMP1 in CRC. Fig. S11. Optimization of guides for targeting the TIMP1 alternative splicing by CRISPR/dCasRx system. [file 13073_2023_1226_MOESM1_ESM.docx]

Supplementary Materials for

**Long-read sequencing reveals the landscape of aberrant alternative splicing and novel therapeutic target in colorectal cancer**

**Sun *et al.***


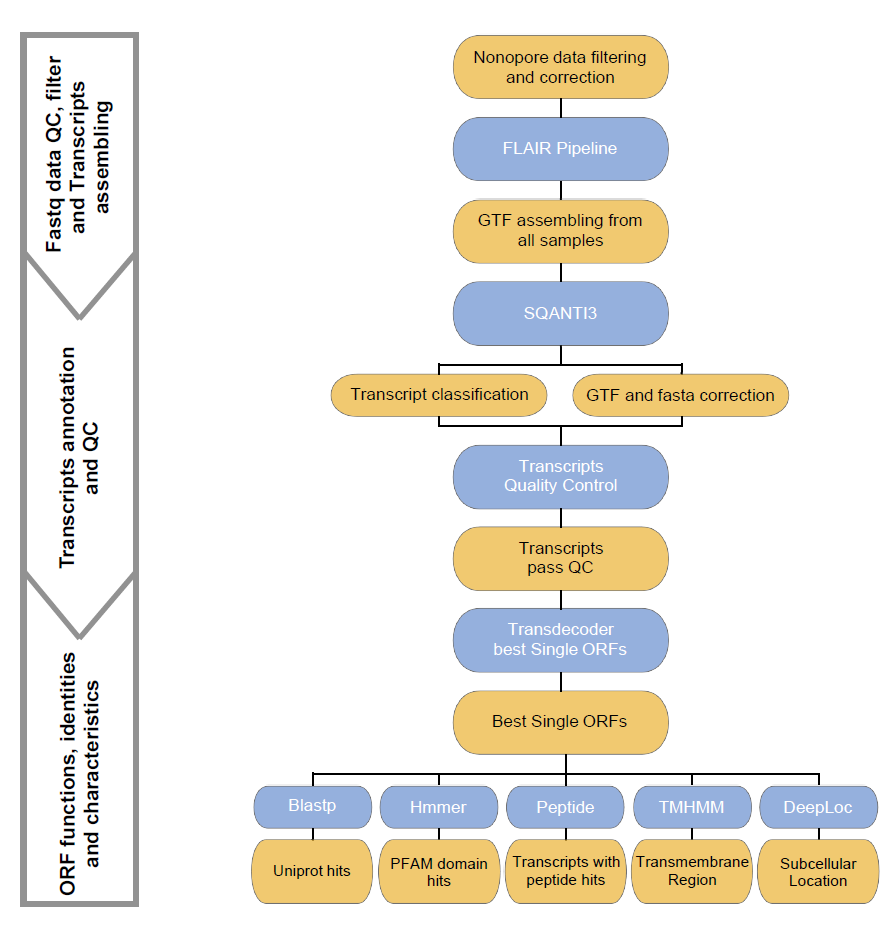


**Fig. S1** **Analysis pipeline for long-read isoform annotation, quality control and prediction of protein features**


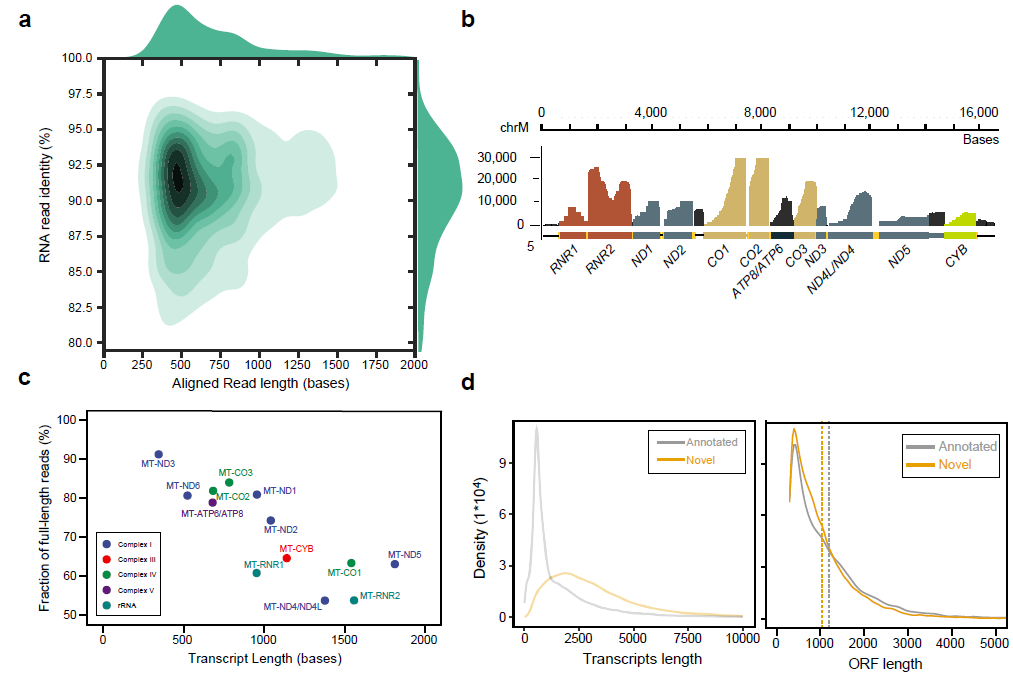


**Fig. S2** **Performance statistics for ONT RNA sequencing.** **a** Alignment identity versus read length for long-read ONT reads. **b** Sequencing coverage of ONT reads derived from the human mitochondrial genome. Labeled colored bars represent protein-coding genes or ribosomal RNA (RNR1, RNR2). **c** Relationship between expected gene read length and factions of full-length ONT reads for individual MT genes. Full-length reads were defined as reads whose 5’ end extended at least within 25 nt of 5’ terminus of the individual genes. **d** Density plot of transcript length (left) and ORF (right) length between novel and annotated transcripts.


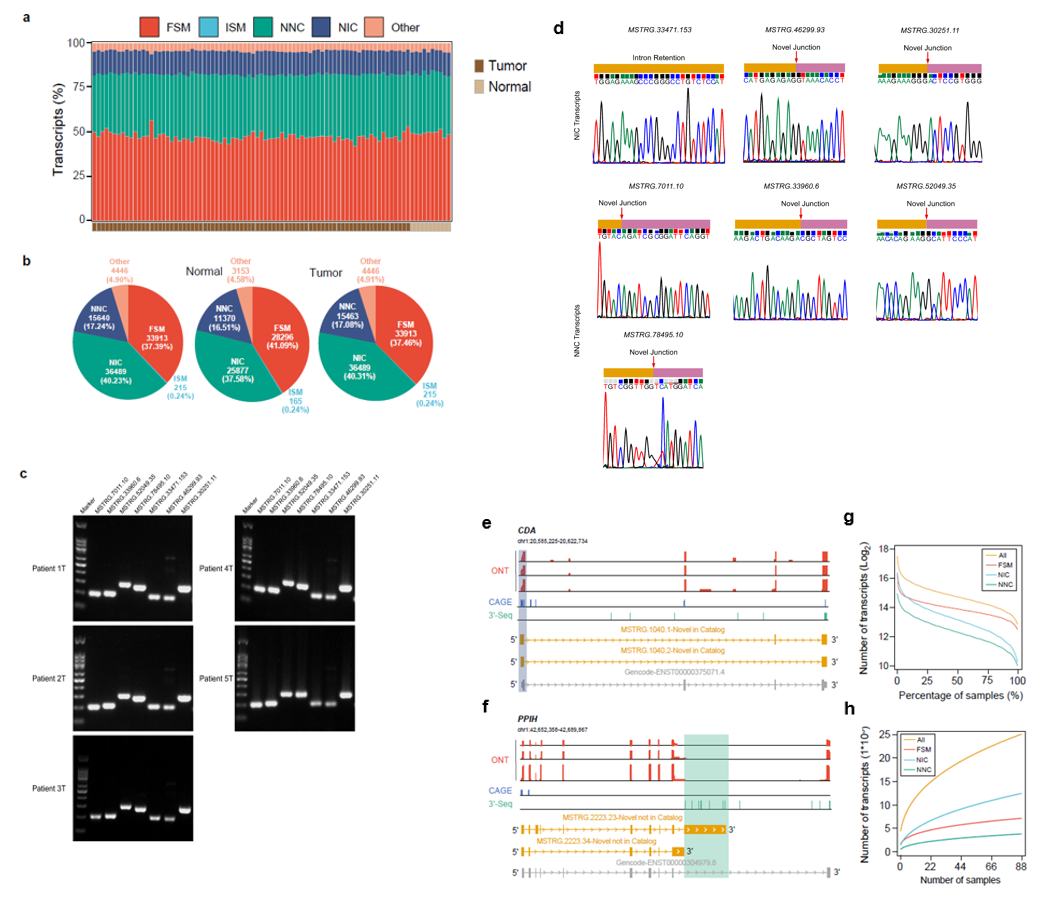


**Fig. S3** **Characteristics of isoforms detected by ONT long-read sequencing.** **a** Percentage of Long-read ONT transcripts supported by Short-Read Illumina RNA-seq in 78 CRC and 10 adjacent normal samples. **b** Classification of Long-read ONT transcripts detected in individual CRC or adjacent normal samples. **c.** Several novel transcripts (*MSTRG.7011.10*, *MSTRG.33960.6*, *MSTRG.52049.35*, *MSTRG.78495.10*, *MSTRG.33471.153*, *MSTRG.46299.93*, and *MSTRG.30251.11*) were validated using qRT-PCR. **d** Several NIC and NNC transcripts (*MSTRG.7011.10*, *MSTRG.33960.6*, *MSTRG.52049.35*, *MSTRG.78495.10*, *MSTRG.33471.153*, *MSTRG.46299.93* and *MSTRG.30251.11*) were validated using Sanger sequencing method. **e, f** Structure of CDA **(e)** or PPIH **(f)** novel NIC or NNC Long-read ONT transcripts compared to GENCODE v34 annotation, along with CAGE peaks supporting the novel TSS **(e)** and 3’-seq peaks supporting the novel TTS **(f)**. Novel TTS or TSS are highlighted. **g** Number of transcripts detected in the percentage of patients. **h** Number of transcripts detected versus number of samples profiled. Annotated FSM transcripts (red) reach saturation, while novel transcripts remained unsaturated.


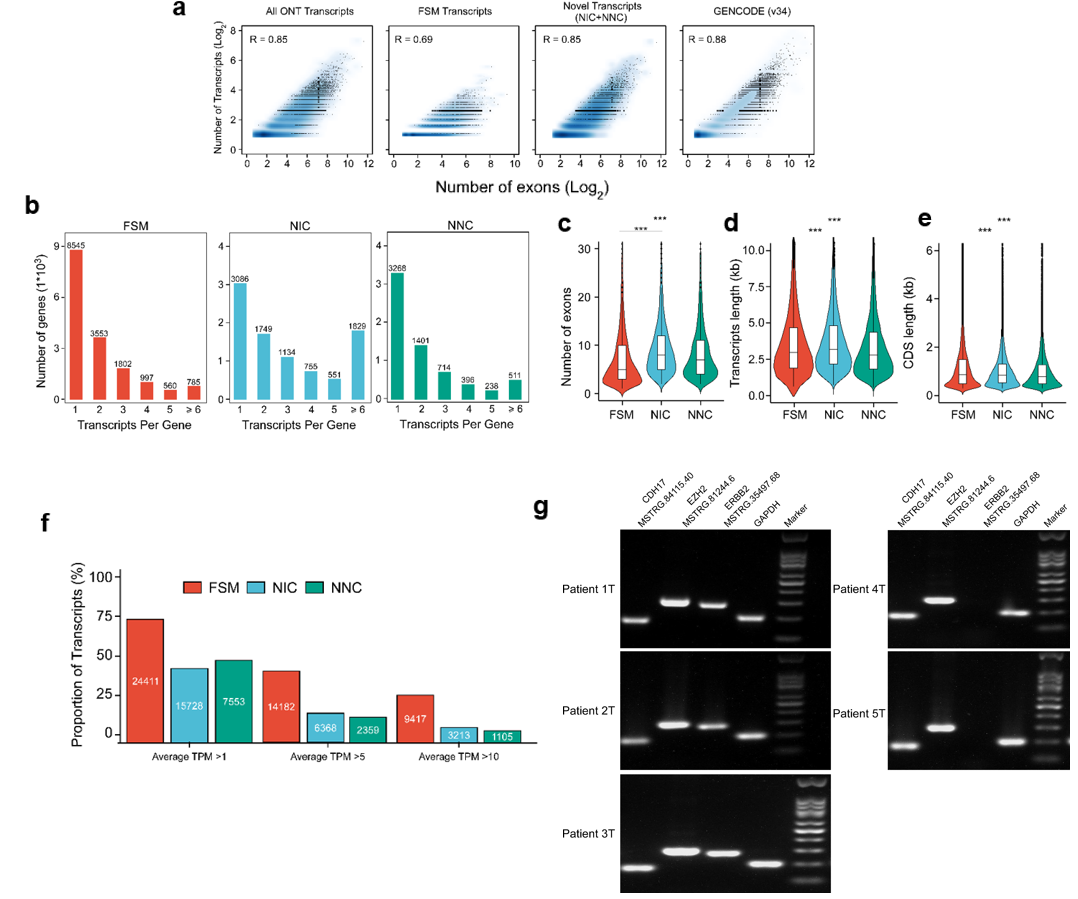


**Fig. S4** **Characteristics of different types of Long read ONT identified transcripts. a** Correlation between number of exons and number of different types of transcripts. **b** Number of different types of transcripts identified per gene. **c** Number of exons of different types of transcripts. **d** Length of different types of transcripts. **e** Length of CDS region of different types of transcripts. **f** The proportion of expressed annotated and novel transcripts identified in CRC samples. **g** Several novel transcript isoforms were validated using qRT-PCR.


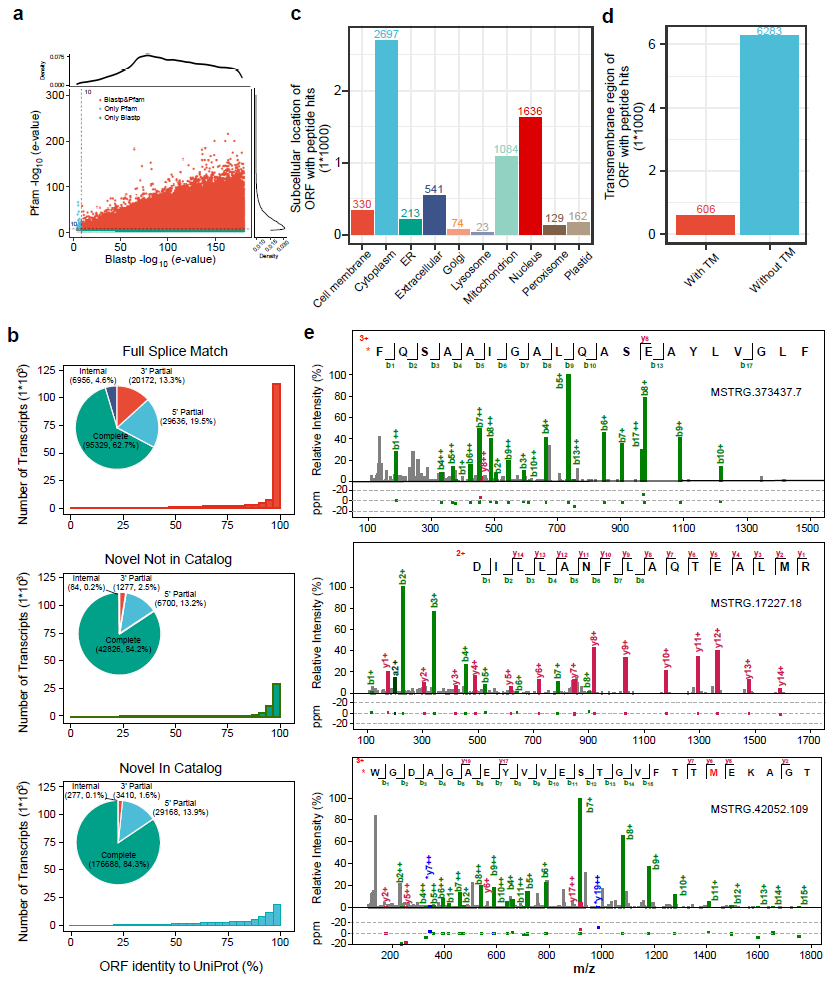


**Fig. S5** **Novel transcripts detected in colon tumors by ONT long-read sequencing are predicted to impact protein sequence, domains, or localization. a** Correlation between blastp e-value and pFAM e-value novel transcripts. **b** Subcellular location of ORFs with MS/MS validation predicted by DeepLoc. **c** Transmembrane domain of ORFs with MS/MS validation predicted by TMHMM. **d** Percentage of amino acid sequence identity for predicted ORFs compared to their closest human protein isoform in UniProt database. **e** Spectrograms of novel transcripts with peptides validated by targeted MS PRM model.


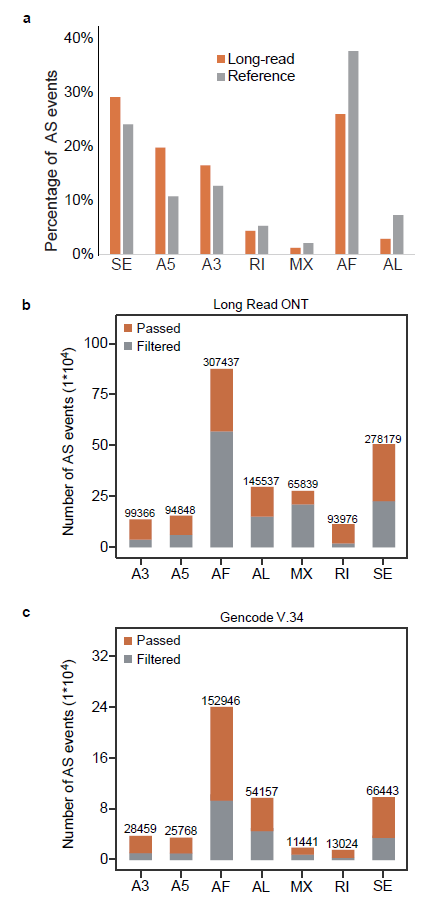


**Fig. S6 Identification of alternative splicing events in CRC. a** Percentage of seven types of AS events (left) or corresponding genes (right) detected in CRC tumors. **b** Number of seven types of AS events identified by long-read sequencing. **c** Number of seven types of AS events identified by Gencode v.34 reference.


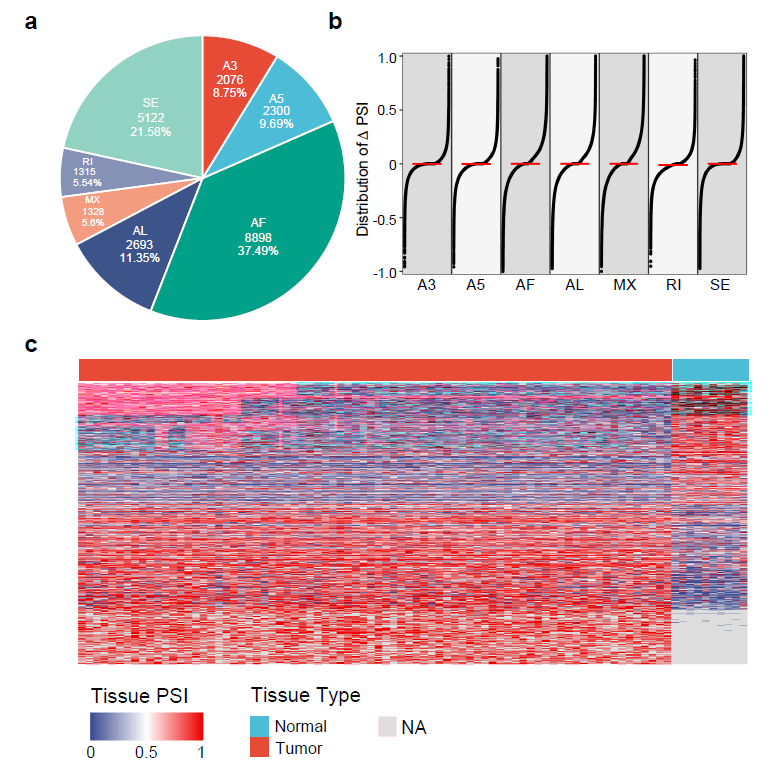


**Fig. S7** **Identification of differentially expressed alternative splicing events in CRC. a** Percentage of seven types of DEAS events identified in CRC. **b** Distribution of ΔPSI of seven types of DEAS events identified in CRC. **c** Heatmap of differentially expressed AS events identified in colon cancer.


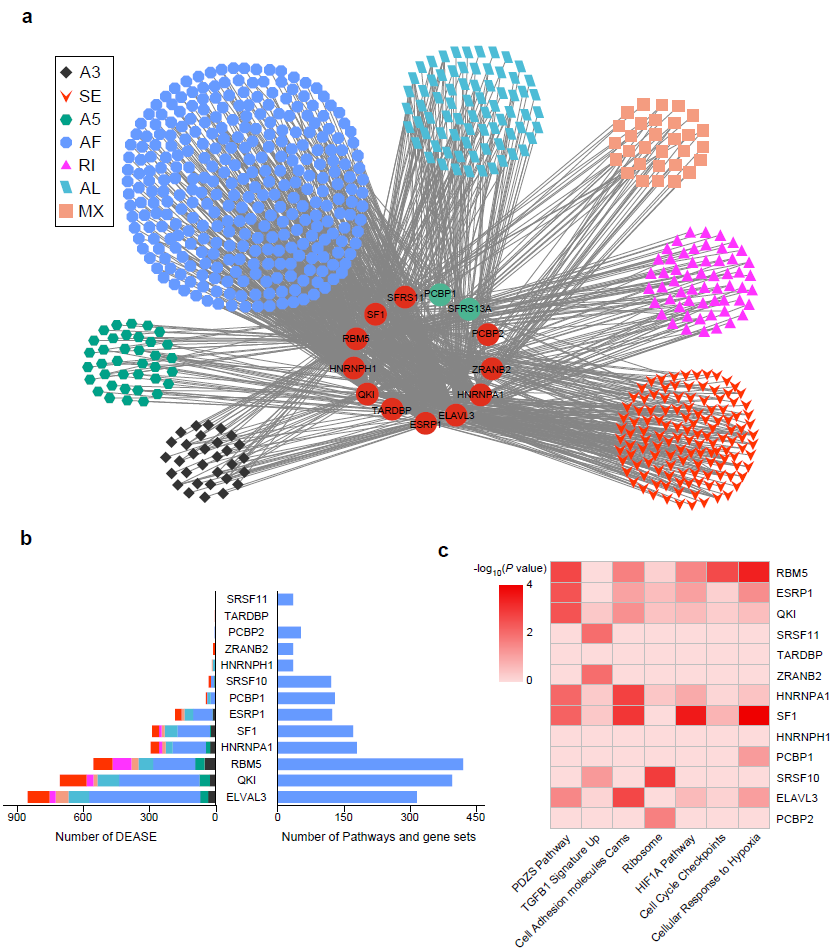


**Fig. S8** **The regulation network of DEAS events by differentially expressed SFs. a** Dysregulation network between DEAS events and SFs. Labeled circles in the center represent SF genes. Red circles denote up-regulated SF genes in tumors, whereas green circles denote down-regulated SF genes. Colored circles connected to RBP genes by gray lines are distinct types of DEAS events. **b** Left panel shows the number of distinct types of DEAS events regulated by each SF, bars in right panel indicate the number of pathways regulated by each SF. **c** Heatmap shows the enrichment of pathways regulated by SFs. Different colors indicate the adjust *P* values (-log_10_ transformed) generated from enrichment analysis.


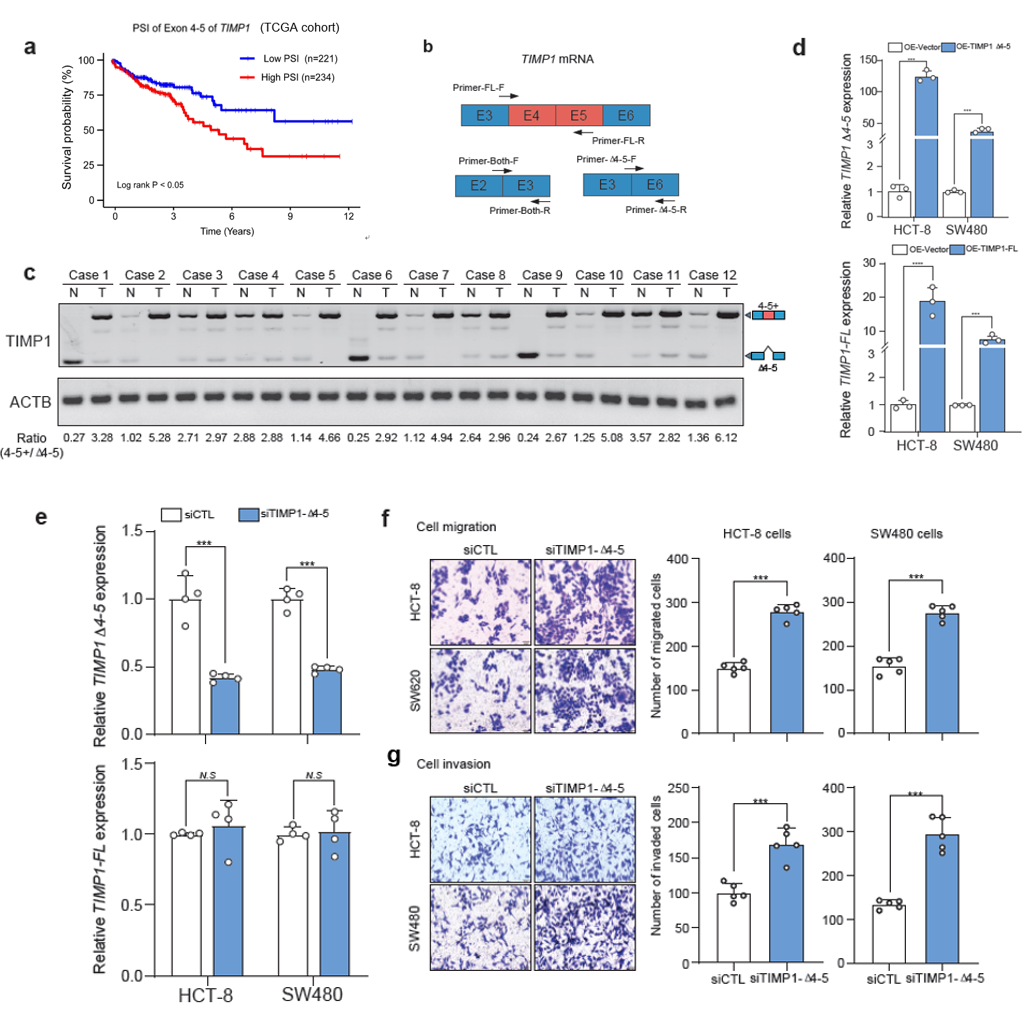


**Fig. S9** **Dysregulation of TIMP1 exon 4-5 splicing in CRC. a** Patients’ overall survival rate based on PSI value of TIMP1 exon 4-5 in TCGA cohort. **b** Scheme describes the qRT-PCR primer design strategy to quantify different transcripts. **c** RT-PCR results depict the ratio of transcripts with or without exon 4-5 in 12 pairs of CRC and adjacent normal tissues. The ratios were quantified by the image J software. **d** qRT-PCR results of TIMP1 Δ4-5 (left) or TIMP1-FL (right) expression in different CRC cancer cell lines after transfecting with empty vector, pCS2-TIMP1-FL, or pCS2-TIMP1-Δ4-5 plasmids. *P values* were calculated by Two-sided Student t test, *** *P* < 0.001. **e** qRT-PCR results of TIMP1 Δ4-5 (up) or TIMP1-FL (bottom) expression in different CRC cancer cell lines after transfecting with control siRNA or siRNA targeting TIMP1 Δ4-5 transcript. *P values* were calculated by Two-sided Student t test, *** *P* < 0.001, N.S, not significant. **f, g** Tumor cell migration **(f)** and invasion **(g)** assay of HCT-8 and SW480 cells with indicated treatments. *P values* was calculated by Two-sided Student t test, *** *P* < 0.001. The migrated or invaded cells were quantified by counting in five fields. Scale bar, 100 μm.


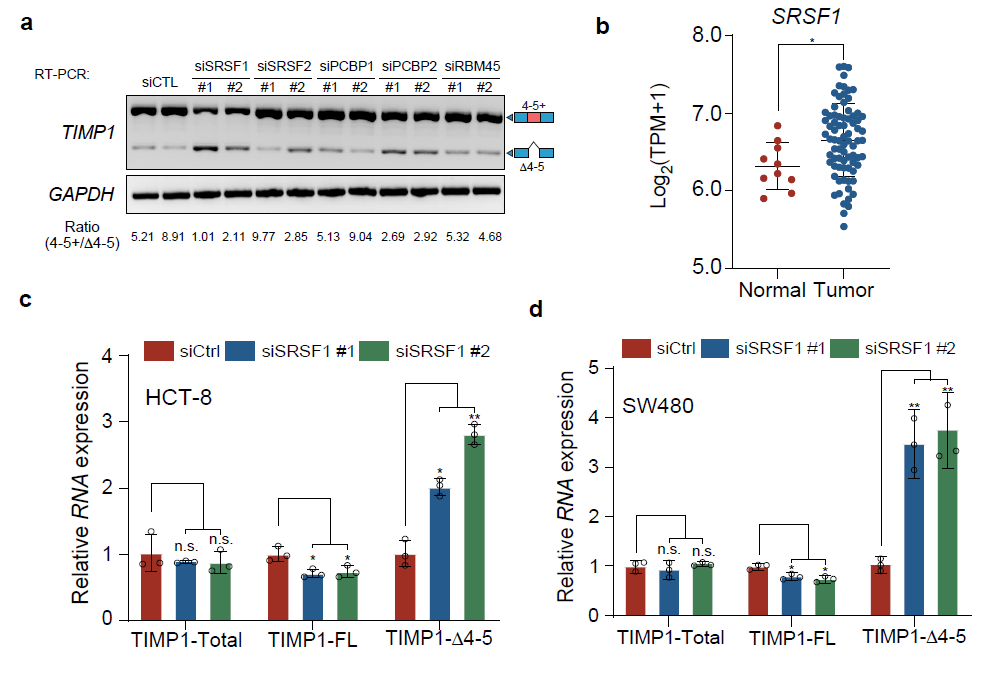


**Fig. S10** **SRSF1 sustains the exon 4-5 inclusion of TIMP1 in CRC. a** RT-PCR results depict the ratio of transcripts with or without exon 4-5 in SW480 cells transfected with siRNAs targeting different splicing factors predicted by RBPMap. The ratios were quantified by the image J software. **b** Expression of SRSF1 in CRC Soochow cohort calculated by Illumina sequencing. *P values* was calculated by Two-sided Student t test, * *P* < 0.05. **c**, **d** qRT-PCR results of TIMP1 Δ4-5, TIMP1-FL, and total TIMP1 expression in HCT-8 (**c**) or SW480 (**d**) cells after transfecting with control siRNA or siRNA targeting SRSF1. *P values* were calculated by Two-sided Student t test, * *P* < 0.05, ** *P* < 0.01, N.S, not significant.


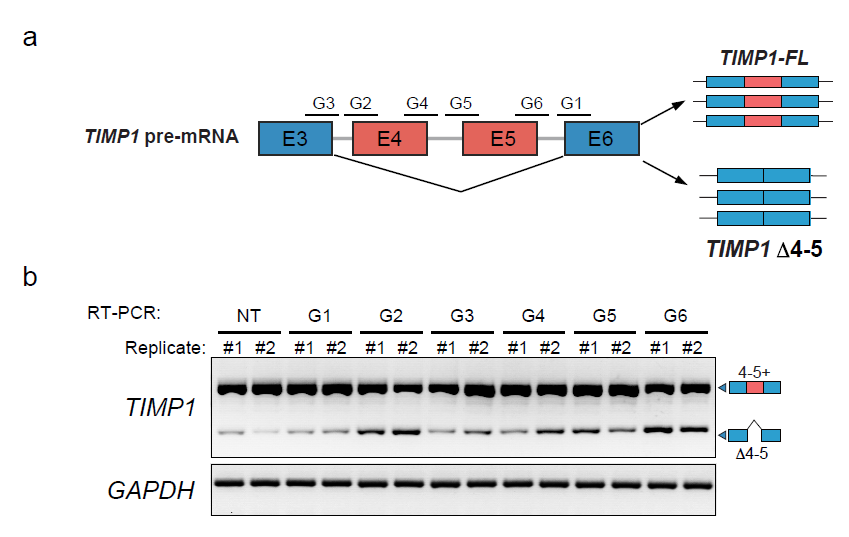


**Fig. S11 Optimization of guides for targeting the TIMP1 alternative splicing by CRISPR/dCasRx system. a** Scheme depicts targeting the TIMP1 alternative splicing by CRISPR/dCasRx system. **b** RT-PCR results depict the ratio of transcripts with or without exon 4-5 in SW480 cells transfected with CRISPR/dCasRx plasmid and different guides.
